# A reflection on participatory research methodologies in the light of the COVID-19 – lessons learnt from the European Research Project TRIPS

**DOI:** 10.12688/openreseurope.14315.1

**Published:** 2021-12-16

**Authors:** Alexandra König, Tally Hatzakis, Alexey (Aliaksei) Andrushevich, Evert-Jan Hoogerwerf, Elvia Vasconcelos, Carolina Launo, Laura Alčiauskaitė, Steven Barbosa, Kristina Andersen

**Affiliations:** 1 German Aerospace Center (DLR), Braunschweig, Germany; 2 Trilateral Research, Waterford, Ireland; 3 AAATE, Linz, Austria; 4 Technical University of Eindhoven, Eindhoven, The Netherlands; 5 TBridge, Bologna, Italy; 6 European Network of Independent Living (ENIL), Brussels, Belgium; 7 UITP, Brussels, Belgium

**Keywords:** accessibility, participatory research, co-creation, COVID-19

## Abstract

The coronavirus disease (COVID-19) outbreak has had considerable impacts on research projects, particularly those adopting participatory approaches. This paper reflects on the methodological adaptations employed by the European research project TRIPS to facilitate co-design and open innovation practices towards the development of accessible mobility solutions. The article reports how the methods were adapted to facilitate participatory research with almost no physical meetings. In doing so, the paper presents the alternative ‘distanced-based’ participatory approaches employed to engage users with disabilities and institutional stakeholders in the transport ecosystem, like online workshops, social media content analysis, online surveys and peer-to-peer telephone interviews. Lessons learnt and practical guidelines for distance-based participatory research are presented and discussed with the aim of increasing resilience in the light of future changes.

## Plain language summary

The novel coronavirus forced people all over the world to avoid direct social contacts. As a consequence, research projects working with members of the public, so called participatory research projects, had to re-think their original methods. The European research project TRIPS was one of these projects that changed their methods as a reaction to the coronavirus pandemic (COVID-19). This article reports how the methods were adapted to facilitate participatory research with almost no physical meetings. The presented methods were assessed by the authors. Based on this assessment, recommendations for other researchers are described. These recommendations might be of help to other researchers conduct studies with a reduced need for physical presence in meetings.

## Introduction

### Challenges for participatory research in relation to the COVID-19 pandemic

When the novel coronavirus (SARS-CoV-2) began to spread in early 2020, governments around the globe implemented social distancing measures to interrupt its transmission ( WHO, 2020a). Along with other facets of life, like childcare and mobility, this has had a negative impact on all participatory research relying on important human interaction and presence for generating new knowledge. Participatory research is characterised by the goal to include members of the public in research, and by “being reflexive, flexible and iterative” ( Cornwall & Jewkes, 1995, p. 1668). Oakley (1991) defined three facets of participation: contribution, organization and empowerment, shifting the focus from research “about people” to research “with people”. Participatory research projects focus on planning and conducting the research process with groups of people whose attitude, choices and behaviour are under study. Consequently, this means that the aim of the inquiry and the research questions develop from the convergence of two perspectives, i.e., science and practice. In this case, both sides benefit from the research process ( Bergold & Thomas, 2012).

Due to the COVID-19 pandemic, governments banned interpersonal interactions that are an essential precondition for participatory research, and could no longer be carried out on-site. This had an enormous impact on user research in different fields, like user needs analysis in the context of transport systems (c.f. König & Dreßler, 2021). In response, participatory research projects had to re-think their original methods and research methodology in various ways. The research project EQUIMOB ( Utrecht University, 2021), for example, postponed the planned field work regarding gender effects and inequalities in mobility options in Asian countries and used telephonic interviews to assess the impacts of the pandemic on mobility behaviour ( EQUIMOB project team, 2020). Online instead of face-to-face interviews were also used by the Children Caring on the Move project, to face the pandemic situation ( Children Caring on the Move, 2020).

A literature review by Hall, Gaved, and Sargent (2021) was one of the first studies that provided an overview of over 38 documents regarding participatory methods within the context of COVID-19. The paper reflects upon the challenges of distance-based participatory research methods, like ethical implications, IT literacy and equal opportunities for engagement. Based on this reflection, the authors derived implications for future projects, like the challenge to include those who do not have access to technologies in distance-based participatory research.

Another recent publication reflects upon stakeholder engagement in participatory marine science projects in the EU ( Köpsel *et al*., 2021). The authors describe coping strategies adopted by 30 projects and recommend seven practical actions to facilitate stakeholder engagement during the pandemic: “1) know your stakeholders (better than before), 2) strengthen existing relationships, 3) do not go 100% digital, 4) re-think your offline methods, 5) stay flexible and keep it simple, 6) apply lessons in post-pandemic engagement, and 7) account for the COVID-19 circumstances in your research results“( Köpsel *et al*., 2021).

The CLIMAFRI project sought to reduce flood risks in Togo and Benin by integrating science-based data with insights from local stakeholders and communities. The project used virtual instead of the physical stakeholder workshops originally planned ( United Nations University, 2020).

Community-based participatory research in the context of HIV research shifted the stakeholder-led steering committee meetings to remote meetings ( Nguyen *et al*., 2020).

The European research project, ART-Forum, had planned interactive workshops with experts to simulate scenarios of autonomous driving ( Interreg, 2021). To facilitate dialog and turn-taking, an online Delphi study was conducted with several iterative runs.

In one of the first scientific papers on the topic of stakeholder engagement in the COVID-19 era, Tobin, Mavrommati and Urban-Rich (2020) reflected on the methodology changes of their research on marine microplastics. They used three-hour online workshops instead of five-hour physical workshops on site. As an adaptation to remote work, they asked the participants to watch expert videos for preparation purposes ( Tobin *et al*., 2020). Reflecting on their experiences, the authors recommended further research in exploring facilitation of online workshops, such as using breakout rooms.

Besides the above, a number of new projects have focused on generating data relating to the effects of the COVID-19 pandemic on different aspects of life, relying on crowdsourcing to collect data. Crowdsourcing is defined by a type of participative online activity implemented by individuals or groups to collect data ( Estellés-Arolas & González-Ladrón-De-Guevara, 2012). The open portal coronarchiv collects personal memories, like diaries, photos or social media chats reflecting life during the coronavirus pandemic ( coronarchiv, 2020). The Corona Data Donation project has collected data like temperature and heart rate from over 500.000 volunteers’ wearable fitness devices ( Robert Koch-Institut, 2020).

### Case study – the European research project TRIPS

The European Research project TRIPS (TRansport Innovation for vulnerable-to-exclusion People needs Satisfaction) developed and applied a participatory research approach to increase accessibility of public transport for persons with disabilities. TRIPS put forward a co-design approach that underpins Mandate 473: Design for All to eliminate discrimination and improve Access for All to mobility services ( European Commission, 2020). The project developed and applied a participatory approach that aimed to 1) co-produce knowledge on existing barriers in transport, 2) co-create solutions for making transport more accessible and 3) co-evaluate the resulting prototypes and services in the seven cities, i.e., Bologna, Brussels, Cagliari, Lisbon, Sofia, Stockholm and Zagreb. The Co-design-for-All methodology creates the conditions for the equal participation of all citizens in open innovation and for the development of inclusive mobility designs from their inception. In doing so, the project addressed the expected impacts of the call to help regional authorities and businesses in designing digital transport solutions that cater for individual needs.

A key methodological output of the TRIPS project was to develop and prove the social value and validity of a co-design-for-all methodology that enables equal access to open innovation to all citizens, including those with disability. To do so, seven pilot case studies were planned, that demonstrated the value of the approach and provided reference examples by applying it in seven European cities.

Consequently, achieving true participation and hands-on involvement was paramount for the project. Hence, attention to achieving and maintaining this focus, despite the complications presented by COVID-19, is a top priority for the project.

**Figure 1.**
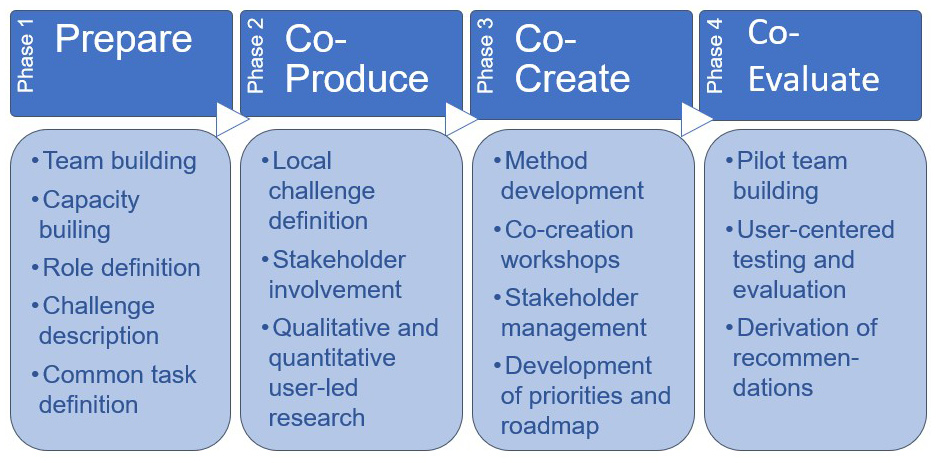
Original methodology of the TRIPS project.

The project started in February 2020 and has a term of three years. At the time of writing this paper, the first two phases of the project were finished (see Figure 1). Phase 3 (“co-create”) is ongoing and will be closed in spring 2022.

## Reflection on participatory TRIPS methodology

### Initial TRIPS methodology based on project description

TRIPS is a participatory case study research (Reily, 2019), in that it actively involved working groups of users and representatives of the transport community in all phases of the research and innovation process, from conceptualising the study to report writing and dissemination. It is ideologically oriented and emancipation-motivated, proposing radical changes in the social processes and innovation structures that shift the balance of power in knowledge production and use, with respect to understanding and responding to users’ mobility needs. So far, disabled users have only nominally been involved in developing mobility innovations. TRIPS sought to emancipate the users to play a central role throughout the innovation process from user research, to prototyping, to business case development. To achieve this, TRIPS brought together 10 partners and working groups in seven European cities (Bologna, Brussels, Cagliari, Lisbon, Sofia, Stockholm and Zagreb) to demonstrate how empowered people with disabilities can play a central role in the design of inclusive digital mobility solutions.

As shown in Figure 1, the methodology of TRIPS was based on four phases of participatory work: 1) preparation, 2) co-definition, 3) co-creation and 4) co-evaluation. The methodological approach of TRIPS drew together diverse methods from a broad range of academic fields, to support open collaborative innovation that engage users and communities ( Baldwin & von Hippel, 2011). The project aimed to extend Wright and McCarthy’s (2015) notion of participatory design, in which they stated that ‘knowing the users” in their day-to-day lives involves understanding what it feels like to be that person and what their situation is like from their own perspective. As such, the project commenced with identifying the gaps between user needs and preferences with regards to existing urban trans port and future mobility trends, as well as institutional and cultural barriers that prevent institutional actors to meet those needs. The project also gauged users’ views on future mobility systems and emerging ICT and assistive technologies that can facilitate users’ interaction. To create common grounds and promote systemic change, it also brought together disabled users and transport and assistive technology experts to co-develop and agree on a common innovation roadmap, common research priorities and joint policy recommendations. These communities work jointly to validate these outcomes with their peers, as well as to extend the validation of their relevance with other NGOs representing other communities vulnerable to exclusion, such as senior citizens and migrants.

### Methodology adjustments as a reaction to the COVID-19 pandemic

One of the main aims of Participatory Design (PD) was the active involvement of all stakeholders as co-designers ( Holone & Herstad, 2013). Given the fact that participation cannot be imposed as a one-size-fits-all approach ( Thiollent, 2011), it is always negotiated by participants in order to become relevant to their current situation in a meaningful and culturally appropriate way. The COVID-19 pandemic can be seen as another situation that demands negotiation of participatory research practices that go beyond physical proximity, so as to maintain the ethos of participation.

The following section presents the aspects of the COVID situation which motivated the changes of the methods employed, the barriers and drivers leading to the particular adaptation choices made in search of maintaining the ethos of the initially intended study. Table 1 lists the objectives of the TRIPS project and compares the original methodologies to the adapted methodologies. As shown here, we drew on the personal strategies employed by people with disabilities to stay in touch remotely, be socially connected while physically distant, together with paying special attention to methods where absence and delays can be considered qualities rather than problems.

**Table 1.** Comparison of the original and alternative methodologies.

Objective	Originally planned methodology	Alternative or adjusted methodology
To empower disabled citizens to take part in research and development and facilitate the research amongst peers with respect to their access needs, mobility requirements and attitudes towards future mobility solutions.	Establishment of user community and working groups, consisting of users with disabilities, transport providers, city authorities, assistive technology suppliers and other parties interested.	The established working groups in seven project cities held remote meetings.
To identify barriers that people with access needs face before, during and after their travelling with public transport	Shadowing of public transport users during their trips and subsequent questioning.	Social media content analysis performed by local user leads
To acquire in-depth information and insights concerning the travel patterns, attitudes and opinions of people with disabilities	Face-to-face interviews by researchers	Online peer-to-peer interviews
To gauge people’s attitudes towards future mobility systems	Online survey to be disseminated during conferences and workshops following a multimedia presentation of the mobility systems.	The survey was run online without the audio-visual presentation and support. Recruiting survey participants relied solely on word of mouth and extensive dissemination
To develop a multi-dimensional metric to measure the accessibility of different public transport systems regarding travel needs, such as timing, comfort, feeling of security of people with disabilities	Focus group workshops to be held online or face to face (originally not defined) involving stakeholders’ representatives	Focus group workshops implemented online and supplemented by an online survey
To review mobility solutions together with stakeholders, and co-develop design concepts for future mobility solutions that are equally accessible, intuitive and friendly to all users.	In-situ innovation workshops in Brussels	Online interactive co-design workshops with people with disabilities delivered in their native languages, for the seven cities
To discuss the institutional barriers to the appropriation and implementation of suggested technologies and discuss potential solutions.	In-situ workshops in seven cities with local stakeholders	Online interactive co-production workshops as flexible units with stakeholders delivered in native languages for the seven cities
To co-create collaborative methods with the seven groups of persons with disabilities working in the project.	A string of in-person activities allowing for the methodological approach to be designed in short bursts of engagement.	A long series of regular 1:1 sessions, where we used a combination of qualitative research methods
To deploy the collaborative methods as peer-to-peer activities in each of the seven cities in the project.	Peer-to-peer in-person activities	Peer-to-peer online activities: one to three whole-group workshops; two to four offline activities.
To generate knowledge, ideas and concepts for improving the mobility of people with disabilities through collaborations between people, technology and society.	Research through design by different methods like workshopping and sketching	Used predesigned kits, including artefacts, maps or photographs for facilitating remote co-creation and creatively sharing concerns and knowledge. Examples are design probes ( Gaver *et al.*, 2004) or bespoke booklets ( Desjardins *et al.*, 2019)

The first objective that underlined the following steps, was to empower citizens with disabilities to take part in the research over the course of the entire project. Originally, it was planned to establish working groups in each of the seven partner cities, consisting of 10 to 15 people with different access needs and local representatives, like transport providers. The local working groups were composed of a local user lead (LUL), who are disability activists and, in many cases, people with disabilities themselves; the core user team (CUT) and representatives of stakeholders. Due to the legal contact restrictions, the working groups did not meet in person but had remote meetings using the online video platforms every four weeks.

Another objective of the project was to identify barriers that people with access needs face when travelling on public transport. To reach this goal, the project description had planned to conduct a shadowing study to observe users taking public transport during their trips in the various partner cities, to understand the challenges they face during their end-to-end journeys and explore the criteria affecting their transport-related decisions. Giving the partially discontinued public transport service when the shadowing study should have taken place and the high risk of infections when using public transport, the shadowing study could not be conducted. Instead, the team decided to pursue a social media content analysis to identify barriers to public transport use retrospectively. Social media content analysis utilises user-generated social media data as a barometer for attitudes regarding specific topics ( Lai & To, 2015). In the context of TRIPS, the social media content analysis provided insights into the discussion regarding public transport use in each city.

A further aim of the project was to acquire in-depth information and insights concerning the travel patterns, attitudes and opinions of people with disabilities. For this purpose, a qualitative interview study was originally planned to be conducted face-to-face. Due to the social distancing measures, the interview study was conducted online using videoconference systems. Each LUL interviewed seven persons from their cities in their native tongue. See Alčiauskaitė, Sioen, Barbosa, Dreßler and König (2020) for more information.

A survey was created to gauge people’s attitudes towards future mobility systems. The survey was conducted online as planned, however, the dissemination approach changed as it was originally planned to use conferences, workshops and webinars to present the mobility systems in a vivid, multimedia way. The consortium had originally planned an online questionnaire, through which we would collect data via online voting during conferences and workshops organised around punctuated events, such as the International Disability Day in December, as well as via our working group members during their personal interactions with other disabled citizens during local meetings. The idea was for partners and working group members to participate in various local events, present a multimedia presentation of the mobility systems allowing users to ask questions in order to understand the mobility concept presented, and then answer a number of questions regarding their intention to use it, the value of such systems and possible ways they would adapt it to better fit their lifestyle. Due to the lack of conferences and workshops on site, the survey was conducted without the audio-visual presentation and support. The recruitment of participants had to rely solely on word of mouth and extensive dissemination ( Alčiauskaitė *et al.*, 2020).

One of the main aims of TRIPS was to develop a multi-dimensional metric to measure the accessibility of different public transport systems, the so-called Mobility Divide Index (MDI, Bagnasco *et al.*, 2021). To determine the index structure, investigate and prioritize the main dimensions that could influence people with disabilities’ travel experience, two workshops were planned and organized online, recruiting the TRIPS local working groups. Furthermore, an online survey that was not originally planned, was conducted to assess and weight the identified dimensions of the MDI from a user perspective (N = 113).

In-situ workshops in Brussels were planned, to co-create concepts of future mobility solutions that help overcome existing barriers. In their place, seven online interactive co-design workshops with people with disabilities were conducted in the corresponding languages of the seven cities ( Hoogerwerf *et al.*, 2021).

Workshops with stakeholders were originally planned for discussing the institutional barriers to the suggested solutions, and to identify facilitators and barriers to their implementation. Because of the pandemic, local needs in each of the partner cities varied and, with this, the design of the final delivery. As a result, the workshops were planned as a series of units that partners could combine for use in one-, two- or three-day workshops of variable length and delivered online. In total, 13 workshops were conducted with a total of 100 participants ( Hoogerwerf *et al.*, 2021).

One of the main goals of TRIPS was to devise a co-design methodology for all, with accessibility principles of engagement and a strong stance on access, participation and ownership. The methodological foundations we built upon tend to prioritize physical presence and face-to-face modes of inquiry. However, in the process of mitigating our strategies to allow the intended spirit and ethos of participation to be conducted without physical presence, we were reminded that people with disabilities have always faced additional barriers to physical mobilization (e.g., Wilson, 2003), as underlined by the main driver of TRIPS: access to public transport for full participation in society and independent living.

As a mitigation strategy, this work was conducted entirely online, through a phased approach that intercalates the execution of tasks and reflecting, where knowledge is generated in a collaborative and iterative manner, and research and action are linked together by critical reflection. This work started with defining a theoretical foundation for participatory inquiry in the context of the current limitations imposed by the pandemic. This established the stage for what constitutes the main ongoing process of the TRIPS methodology: to co-create collaborative methods with the seven CUTs of persons with disabilities working in the project. During this process, each cities’ LUL and LC were engaged to create localized versions of the methods online, which were subsequently deployed as peer-to-peer online activities in each city. The first phase of deployment took place from March to May 2021, where each city was engaged in a series of activities aiming to formalize their unique identity as a group and their vision for what they wanted to achieve within the duration of TRIPS. This work was done in preparation for subsequent work to develop local mobility solutions in collaboration with institutional actors. The outputs of this deployment constituted the first iteration of the methods co-created and customized to the concerns and identities of the seven CUTs in the project. The next iterations of deployment and reflection will focus on the experiences of using these methods in practice, culminating in the end of the project as lessons learned, general principles, evaluation data and application guidelines.

### Reflection on participatory methodology adaptation in TRIPS

In the following, the effectiveness, feasibility and goal-reaching capacity of the applied method are reflected on. In doing so, pros and cons of each method are discussed.

### Empowerment of citizens and building of working groups

In TRIPS project, users with disabilities take a central role in the co-design process, not only as passive observers and reviewers but as the individuals initiating the changes and proposals for future mobility solutions. However, building and maintaining relationships remotely has been a very challenging task. The COVID-19 pandemic-adjusted methodology required strong leadership skills from all LULs, as they were the ones building up the local working groups and ensuring that the co-design process was being implemented in all project cities. All of them reported that the pandemic restrictions made it extremely difficult for them to ensure a smooth running of the working group due to various reasons: not all people with disabilities are comfortable using digital technologies; approaching transport providers or city authorities online was complicated; virtual meetings seemed less attractive than offline ones, among others. On the other hand, virtual meetings gave them an opportunity to be more flexible with arranging the meetings, making them easily accessible to more people and reducing logistic effort and cost.

### Peer-to-peer interviews

Conducting the interview study online and based on a peer-to-peer approach entailed various advantages and disadvantages. One of the prevailing advantages was the familiarity and intimacy that was created by the peer-to-peer interview setting as has been reported in earlier studies in facilitating critical inquiry ( Peck *et al.*, 2021; Schmidt, 2017). Presumably, the familiar and trustful atmosphere enabled a greater openness from interview partners and correspondingly more in-depth information. Conducting the interviews in their native language was another advantage of the method changes. The originally planned implementation in English by the project team researchers would have increased language barriers. Regarding the disadvantages, online interview studies face sampling issues due to the selection of interview partners that have access to, and sufficient competency in, these digital systems ( Duffy *et al.*, 2005). For this interview study, the selection of interview partners by LULs in their network most likely affected the representativeness of the sample. The same can be assumed of the interview partners based on the need for digital competency (literacy).

### Social media content analysis

The social media content analysis revealed itself to be a method that produced a large number of insights into the thoughts and attitudes of persons with disabilities regarding their daily mobility challenges (see Alčiauskaitė *et al.*, 2020). However, conducting the study was somehow challenging, as the method was new to the project team as well as the LULs conducting the research. Detailed instructions in the form of a step-to-step manual were essential for guiding the procedure and were thus developed by the project team. Some of the LULs who conducted the social media search faced difficulties in reaching the minimum number of 30 social media entries, whereas others achieved the limit more rapidly. This implies that in addition to the number of social media posts per city, the ability of people to search online, access the websites and use appropriate search terms also differed. They were not all familiar with social media and nor had access to all of the relevant platforms. Thus, accounts were created to enter the social media platforms. To conclude, the social media content analysis proved to be a valuable and feasible method to identify mobility barriers that are discussed in a specific geographic context. However, the quality of the social media content research strongly depended on the digital skills of the people conducting the research. Thus, people conducting the research should be trained to use different social media platforms and to select appropriate search terms.

### Virtual co-design workshops

The adaptation of the co-design process to online activity required pilot co-design workshops to test the suitability of digital tools and the planned methodologies. Thus, for each co-design session format (co-creation of mobility solutions and identification of institutional barriers) a pilot workshop was conducted. The aim of the pilot workshops was to train the seven co-design workshop facilitators, who were often people with disabilities, mostly without many experiences in organizing and conducting workshops. As a training result, the local facilitators conducted the workshops with participants mainly from their own city (Bologna, Brussels, Cagliari, Lisbon, Stockholm, Sofia and Zagreb). The pilot training workshops were very helpful, as their subsequent assessments revealed several recommendations for the workshops in the seven cities:

- The aims and purposes of the workshops should be clearly stated at the beginning
- A guide for the local facilitators to explain how to deliver the workshop would be beneficial.
- The material (hand-outs and work sheets) should be shared beforehand to give the participants the opportunity to familiarise themselves with the content
- Additional guidance for facilitators as footnotes would help in the delivery
- Pictures/figures should be explained to increase accessibility for visually impaired users
- The content on the presentation slides should be reduced
- The time allocated should be adapted to allow more time for discussion of innovative concepts
- Relate the exercises to each other and the bigger picture
- If possible, during the exercises, display a timer counting down the available time
- During the exercises, repeat the question/task every two minutes or give an additional prompt
- Break-out rooms are essential for facilitating discussions in smaller groups
- Virtual warm-ups are essential to replace face-to-face small talk
- After each exercise, allow time for participants to share their answers to make it more interactive
- Remind people periodically to state their name when starting to speak

As a result of the feedback in the pilot workshops, several actions were taken. For instance, all content was made accessible according to best practice guidelines, like the Accessible Online Event Toolkit of the European Disability Forum. Content was also revised to streamline running time and enhance productivity. In addition, a guide for facilitators was produced, alongside delivery notes in the slide deck to facilitate the local workshops. Furthermore, each local facilitator was matched to a project team member to help prepare the workshops and obtain further feedback to guide facilitators and those supporting the workshops. Finally, a more comprehensive range of innovative design concepts was incorporated to represent the variety of user needs and potential solutions.

Despite the fact that a pilot training workshop was conducted for both workshop formats, the actual workshops in the seven cities faced several challenges. First of all, the importance of shared understanding was emphasized. The participants suggested that the briefing document, which was shared beforehand, should introduce the mobility concepts that were addressed during the workshop to reflect upon them before the workshops and thereby increase creativity during the workshops. Another suggestion related to the role of the workshop facilitators, who found it difficult to lead the workshop and to share the power point presentation at the same time. Thus, they proposed to have a person who only shared the power point presentation and acted as a host during the online meeting and admitted people into the online conference.

It was further shown that although most participants had some experience contributing in online workshops or meetings, the required level of interaction and focus was more intense than for *in-situ* workshops. Thus, it should be recognised that participants' capacity to fully contribute in an online forum is considerably lower than in physical settings. The assessment of the online workshops also emphasises the need to consider levels of eyestrain, discomfort, and attention in more detail for the future.

### Online survey

COVID-19 deprived us from this interpersonal interaction preceding the survey as all physical events, conferences and workshops were cancelled, and even online events were postponed. Therefore, we tried to replicate such interactivity online. Initially, we tried to source and include existing videos explaining such systems, but licence-free videos were mostly used for promoting specific brands. Moreover, most videos were only available in English, which is problematic as most citizens approached through the study don’t understand English to a sufficient degree. Finally, demonstration videos are not accessible. Many had videos and textual information, but not voice overs or subtitles. When they had subtitles, they were in English only or their native language and English. Finally, as most videos were on YouTube or corporate websites, linking the survey to these platforms via weblinks meant that the users were directed to external websites, from which they needed to return manually back to the survey. This was difficult for most disabled users who lack the training required to work with information and communication technology (ICT), but particularly difficult for blind people. As a result, we had to rely on a typical online survey format, describing the key components of each without orienting the user. To ensure that the survey was clearly understood, we designed the questionnaire in consultation with the leaders of our working teams and pilot-tested the translated versions with the members of our working groups.

Finally, due to the COVID-19 pandemic, instead of reaching out to users on particular events we set out and anticipated dates and meetings of working group members with their peers locally. To facilitate data collection, we relied on extensive online dissemination via the European Network on Independent Living ( ENIL) membership, disability-related NGOs (European Disability Forum, the International Disability Alliance, Inclusion Europe) and word of mouth. This resulted in a longer data collection period and perhaps a bias towards this section of the population with access to technology and sufficient skills to complete an online survey.

### Online focus group workshops

Two online focus group workshops were performed with the project LULs to achieve our objective of investigating and prioritizing the main variables influencing people with access needs, during their daily travels on public transport. These variables would constitute the core structure of our Mobility Divide Index (MDI).

Since the target audience were people with different impairments, it was essential for us to prepare the material well in advance, using simple words and applying easy-read techniques to make presentations accessible for all, especially for people with reduced vision.

The size of focus groups is generally recommended to be between seven and 10 participants. Considering the complexity of the topics covered in our workshops, after a preliminary introduction and discussion with all participants, we choose to divide them into three focus groups (i.e. three concurrent breakout rooms) of three to four people. Each group was tasked with reflecting on a limited set of aspects of their daily journeys on public transport. This choice allowed us to better manage the online sessions and derive more insights.

Since online focus group workshops can be conducted anytime and anywhere, sessions must necessarily have a limited duration, in order to avoid participants getting bored and distracted.

Therefore, we planned sessions of two hours each. In order to respect the planned time slot, we narrowed down the discussion guide to a few key topics. This influenced our study: while we carried out a deep investigation of the main issues that affect people with disabilities’ daily mobility routines, we did not examine prioritization of these issues as thoroughly.

We then made the most of this shortcoming by launching a survey aiming to collect the importance given to the different MDI variables agreed during the online focus groups ,by European people with different access needs.

As mentioned for the mobility survey (see ”Online Survey”), due to the COVID-19 situation, instead of reaching out to users and promoting our survey at specific physical events, we disseminated it online via ENIL channels and contacted disability NGOs representing different access needs. The latter required a great commitment from the members of the respective organizations and resulted in a longer data collection period to reach a significant number of respondents.

### Development and application of co-design methodology

The work to co-create and deploy collaborative methods with the seven groups of persons with disabilities was initially intended to be developed through a string of in-person activities allowing for the methodological approach to be designed in short bursts of engagement. However, since the entirety of the project so far has been conducted online and from home, this work has taken on a much more elaborate and personalized form. To make up for the loss of in-person activities, we engaged each group in a string of conversations to anchor the methodologies into strongly-held local concerns, and to guarantee that the processes remained within our understanding of co-design and co-production, despite the clear limitations of online work. This work unfolded as a long series of regular one-on-one sessions, where we used a combination of qualitative research methods: semi-structured interviews, open-ended activities, writing exercises, surveys, offline activities, etc. Our focus was on creating a dynamic working rhythm and generating mechanisms to allow for heterogeneous interests and in-depth understandings to come forward. From October 2020 until May 2021 each city was involved in: 10 to 16 one-to-one sessions; one to three whole group workshops; two to four offline activities. In the one-to-one sessions, we regularly had two to four participants, and the workshops were open to the full local team (CUT) in each city. The number of activities varied, as in this work we recognize that not all cities arrive at this process on the same footing. Their needs, wants and challenges are unique and contingent to their local contexts, and therefore require ways of working that emerge from within each of the groups involved: specifically, some of the cities require more regular meetings with the project team and closer monitoring, whereas others practice a more independent working style.

 This approach has allowed us to tailor each interaction to local and personal preferences. This may mean that not everyone had the exact same experience, but that we worked towards shared understandings and convergence through an array of interactions and strategies. In practice, this work was done using the following techniques:

- Workshopping: Through workshopping we aimed to create an experience where individuals' narratives coexist with complex understandings of collective knowledge, leading to a great diversity in outcomes.
- Brainstorming: Brainstorming allows for a broad range of knowledge to manifest, be shared and co-created. This has a dual effect in user involvement: it generates possibilities and equally improves the social dynamics of exchange as a basis for shared meaning.
- Sketching: Through sketching we aimed to explore notions of collaborative visual thinking, in which nonverbal techniques like drawing are used to represent unified action.
- Interviews: Interviews elicit individual knowledge and narratives. We proposed to use them as open engagements where personal stories guide participants and interviewers in the narration of lived experience.

For the practical purpose of working within COVID-19 restrictions, these methods were re-purposed in order to be executed online and in smaller groups. To identify local concerns and establish a collaborative atmosphere, we engaged in a string of iterative conversations that made use of elements from brainstorming and interviews, in order to identify a local focus. These conversations were documented as field-reports and through sketches resulting in a consolidated output for each city, together with shared resources for all CUTs. These local outputs were then used as material for workshops, deployed through a number of local iterations that continue throughout the project.

### Software adaptations

Instead of meeting physically, videoconference systems were used to meet within the project team, the project team with the CUT and the seven CUT among themselves, in what became a longer one-to-one process for specifying and establishing specific local challenges and work methods. These online tools generated advantages, e.g., it was easier to stay in touch without traveling, and disadvantages, e.g., it became clear that creating engaging activities was much harder and the potential for misalignment was higher. In addition, these online interfaces come with their own accessibility issues, which influenced the outcomes in part (e.g., group work and in-depth discussions were made more difficult in those circumstances) and forced us to work in much smaller groups.

To communicate the practical software setup, the groups needed to participate in an online session, and we therefore created an access needs protocol. This protocol was intended to be used for all project related work, as well as any other activities that a group is invited to attend, e.g., a meeting with the city council. To create this protocol, we followed Sandra Lange’s ‘Access Rider Exercise’ prompting each group to articulate what they would need, both individually and as a group to fully engage in online activities ( Lange, 2020). The access needs protocol was meant to be used as a way for each group to create and occupy a shared online space whilst actively shaping their interaction conditions in that space. 

Moving to a digital working space also raised a greater need for establishing online collaborative working processes that catered for varying levels of digital skills. Each city required different levels of support and tended to elicit unique working dynamics. This digital setup did not reflect the ways most groups involved in the project normally work, e.g., having a shared folder with up-to-date documents was a surprisingly hard task. The impetus for having online documents has come not only as a way for producing deliverables, but also as the only way to document and share knowledge between the CUTs and the partners involved in the project. In other words, the TRIPS project was set up to work in a digital way, but we need to pay continuous attention to guaranteeing that these online spaces are truly shared environments. Ultimately, creating efficient and productive online working methods in a multiple-partner project requires a significant amount of effort and is a point of ongoing attention.

## Lessons learned and derived recommendations

Several lessons can be derived the assessment of the applied methods. The following nine practical recommendations present a guide for future participatory research projects that face challenges in conducting research *in-situ*.

The need for piloting new methodologies: Have a pilot of online workshops with “real” participants to facilitate the adoption of the method. We found it was absolutely worth the time.

Absence as a feature: Try to value the other side of the impeded cooperation on-site. There are also potential advantages to distance, such as increased time for reflection, broader participation and improved attention due to the sharing of documents and the joint and simultaneous processing of documents and tasks. 

Mixed presence: Be open to experiment with new ways of being together at a distance. Mixed presence might create meaningful exchanges.

Personalised and localised: These new ways of working together will potentially allow us to tailor each interaction to local and personal preferences. This also means that a shared understanding and convergence through an array of interactions and strategies must be facilitated.

Advantages and disadvantages for equity issues: On the one hand, virtual participatory methods like online workshops expand the reach of the research and thus facilitate the participation of vulnerable-to-exclusion citizens, who would otherwise have not participated in *in-situ* workshops, like people living in the suburbs or rural areas. On the other hand, virtual implementation of participatory methods excludes other groups, like persons with low digital literacy.

Stay connected: It is recommended to stay connected with the participants after virtual workshops to ask if they have further inquiries. Virtual communication methods like e-mails can be used to ask participants for an evaluation of the method and further suggestions. Thereby, subsequent ideas can be included in the process and a continuous participation facilitated.

Train co-researchers to apply the methods: Give consideration to the fact that most of the participants are not familiar with methods like conducting co-creation workshops. Thus, local facilitators should be trained and participate at a pilot workshop to experience the methods. It is further recommended to hire an assistant as a workshop moderator who supports the management of participants log-in or the slide show.

Online methods require more focus as they are highly demanding for participants’ attention: Conducting creative workshops online instead of on-site requires more focus and smaller-scale formats, like meetings and workshops, as maintaining attention in online settings is difficult.

Stay flexible: Participatory approaches are inherently flexible and need continuous adaptations. Beyond that, ever-evolving situations, like a pandemic, require dynamic adaptations of research scopes and methodologies. Maintaining flexibility while accepting limitations can ensure the quality of the process.

To summarize, the assessment of the methodological changes and adaptions during the TRIPS project showed us that there are manifold opportunities to deal with challenging situations. We should not only perceive disadvantages but also value opportunities. Thus, these changes generated several benefits: 1) workshops were carried out virtually in seven cities in local languages, and hosted mainly by the LULs and CUTs of the seven cities instead of conducting only one joint workshop in Brussels, 2) accessibility of formats was increased by facilitating the participation of people that would have otherwise not been able to attend and 3) the participants cohort was more diversified.

## Next steps and further work

The reflection on the methodological adaptations for the TRIPS projects as a reaction to the pandemic leaves several unanswered research questions that need to be further considered in research and practice. First, given the fact that participatory approaches rely on trust, continuous rapport and exchange, an emerging research question deals with the issue of facilitating trust building, and a joint working spirit and productive atmosphere in the light of social distancing. What are possible ways to facilitate a trustworthy working atmosphere with digital and non-digital methods? How to replace valuable small talk and networking during workshop breaks of workshops and events when they are conducted online? How to give local teams a sense of ownership? How can a structure that nurtures ownership and governance of working teams?

Further research should also address how virtual methods should be adjusted to the needs of different users. Furthermore, from a perspective of inclusivity, it seems to be worthwhile to find ways to implement easy-to-read material in virtual conferences and workshops.

Already before the pandemic, projects have been implementing participatory research methods for remote work when it was difficult or impossible to work with participants in a co-located context. Exemplary methods are cultural probes ( Gaver *et al.,* 1999). These methods should be considered in the light of the COVID-19 pandemic.

The TRIPS project will continue to implement methods of participatory research. The next step of the project will be the engagement of local users and institutional actors in 1) co-creating prototypes of future mobility solutions, 2) organising user testing of the prototypes and evaluation of the co-creation process, 3) developing the prototypes into local pilot demonstrators, 4) organising local user testing of local pilot demonstrators, 5) conducting business analysis of the local transport ecosystem and 6) developing the business case for the full-scale deployment of the local pilot demonstrators.

Another further step of the project will be the engagement of people with disabilities, public transport operators and institutional actors in developing and validating policy recommendations, research priorities and an industry roadmap for the mobility sector Specifically, the TRIPS project consortium will validate the described design concepts and priorities as well as the derived policy recommendations, industry roadmap and research priorities with users and institutional actors from the seven cities.

## Conclusions

The reflections on the adaptations of the participatory research methodology for the TRIPS project contributes to enabling scholars to be more prepared for conducting participatory research during the ongoing and future pandemic. Keeping in mind that the adjustments caused by COVID-19-related restrictions are going to persist to some extent in the future (e.g., more people are working from home, online workshops replacing face-to-face workshops, Thombre & Agarwal, 2021), it is important for researchers to adapt their research design to the existing situation and turn the limitations into opportunities. This paper contributes to a better understanding of the functioning of remote participation and which of its aspects could be implemented in the future. Moreover, reflecting on the methodology changes showed that opportunities to deal with the new situation are manifold, and participatory research should not see consider disadvantages but value the derived opportunities. To conclude, the methodology reflection for the case study of the TRIPS project provides lessons learned from implementing research during the COVID-19 pandemic but also for other possible future changes and challenges.

## Consent

Written informed consent for publication of the participants details and/or their images was obtained from the participants//parents/guardian/relative of the participant

## Data availability

### Underlying data

No data are associated with this article.

### Extended data

Zenodo: Research material TRIPS, https://doi.org/10.5281/zenodo.5752461 ( König, 2021)

This project contains the following extended data:

- Guideline_briefing document_ media content analysis.docx (Guidelines for conducting the social media content analysis for the CUT, comprising a detailed description of the steps to perform the task)
- Interview guidelines.docx (Guidelines for conducting the peer-to-peer interview study containing semi-structured interview questions and instructions for the interviewer)
- TRIPS Workshop Design.docx (Short description of the workshop design for the co-design workshops)
- TRIPS_ Guidance document for Co-Design Workshops.docx (Guidance document for the co-design workshops, including detailed instructions for the recruiting of participants, structure and tasks of the workshop, accessibility requirements and technical instructions)

Data are available under the terms of the Creative Commons Attribution 4.0 International license (CC-BY 4.0).
