# A reflection on participatory research methodologies in the light of the COVID-19 – lessons learnt from the European Research Project TRIPS

**DOI:** 10.12688/openreseurope.14315.2

**Published:** 2022-03-29

**Authors:** Alexandra König, Tally Hatzakis, Alexey (Aliaksei) Andrushevich, Evert-Jan Hoogerwerf, Elvia Vasconcelos, Carolina Launo, Laura Alčiauskaitė, Steven Barbosa, Kristina Andersen

**Affiliations:** 1 German Aerospace Center (DLR), Braunschweig, Germany; 2 Trilateral Research, Waterford, Ireland; 3 AAATE, Linz, Austria; 4 Technical University of Eindhoven, Eindhoven, The Netherlands; 5 TBridge, Bologna, Italy; 6 European Network of Independent Living (ENIL), Brussels, Belgium; 7 UITP, Brussels, Belgium

**Keywords:** accessibility, participatory research, co-creation, COVID-19

## Abstract

The coronavirus disease (COVID-19) outbreak has had considerable impacts on research projects, particularly those adopting participatory approaches. This paper reflects on the methodological adaptations employed by the European research project TRIPS to facilitate co-design and open innovation practices towards the development of accessible mobility solutions. The article reports how the methods were adapted to facilitate participatory research with almost no physical meetings. In doing so, the paper presents the alternative ‘distanced-based’ participatory approaches employed to engage users with disabilities and institutional stakeholders in the transport ecosystem, like online workshops, social media content analysis, online surveys and peer-to-peer telephone interviews. Lessons learnt and practical guidelines for distance-based participatory research are presented and discussed with the aim of increasing resilience in the light of future changes.

## Plain language summary

COVID-19 forced people worldwide to avoid direct social contact. Consequently, research projects working with members of the public, i.e., participatory research projects, had to re-think their original methods of engagement. The EU-funded H2020 research project TRIPS, was one of these projects that changed ist methods to react to the coronavirus pandemic (COVID-19). This paper reports how the methods were adapted to facilitate participatory research without physical meetings. In addition, it assesses the presented methods and makes recommendations for other researchers conducting similar studies under similar conditions.

## 1. Introduction

According to the motto of the International Day of Persons with Disabilities of the United Nations in 2004 “ *Nothing About Us Without Us!*”, people with disabilities request to be involved in decision processes that affect them ( United Nations, 2004). Accordingly, research has participatory research methods in various domains such as health ( Nicolaidis *et al.*, 2015), child development ( Wickenden & Kembhavi-Tam, 2014) and transport ( Whitzman *et al.*, 2013) to support the collection of more relevant and more valid data. Besides many advantages for the quality of research and the validity of outcomes ( McDonald & Stack, 2016), participatory research and inclusive participatory research in particular are faced with a variety of challenges, among them ethical challenges ( Iacono, 2006), role ambiguity ( Vega-Córdova *et al.*, 2020) and costs, especially for disabled co-researchers ( Vaughan *et al.*, 2020). Adding to this, participatory research is additionally challenged since the spread of the novel coronavirus (SARS-CoV-2) in early 2020, when governments around the globe implemented social distancing measures to interrupt its transmission ( WHO, 2020a). The aim of the paper is to reflect the changes and challenges in the planning and implementation of participatory disability-related research due to the coronavirus pandemic. The paper thereby reflects the experiences made in the European research project TRIPS.

### 1.1 COVID-19 pandemic: Challenges for participatory research

COVID-19 negatively impacted participatory research relying on human interaction and presence for generating new knowledge. Participatory research relies on engaging members of the public in research and by “being reflexive, flexible and iterative” ( Cornwall & Jewkes, 1995, p. 1668). Oakley (1991) defined three facets of participation: contribution, organization, and empowerment, shifting the focus from research “about people” to research “with people”. Participatory research projects focus on planning and conducting research with people whose attitudes, choices, and behaviour are under study. Consequently, this means that the aim of the inquiry and the research questions develop from the convergence of two perspectives, i.e., science and practice. In this case, both sides benefit from the research process ( Bergold & Thomas, 2012).

In response to COVID-19, governments restricted face-to-face meetings that were an essential precondition for participatory research and could no longer be carried out on-site. These restrictions had an enormous impact on user research, like the analysis of user needs in the context of transport systems (c.f. König & Dreßler, 2021). In response, participatory projects had to re-think their original research methods and methodology in various ways. The research project EQUIMOB ( Utrecht University, 2021), for example, postponed the planned fieldwork regarding gender effects and inequalities in mobility options in Asian countries and used telephone interviews to assess the impacts of the pandemic on mobility behaviour ( EQUIMOB project team, 2020). The Children Caring on the Move project used online instead of face-to-face interviews ( Children Caring on the Move, 2020).

A literature review by Hall *et al.* (2021) was one of the first studies that provided an overview of over 38 documents regarding participatory methods within the context of COVID-19. The paper reflects on the challenges of distance-based participatory research methods, e.g., ethical implications, IT literacy, and equal opportunities for engagement. Based on this reflection, the authors derived implications for future projects.

Another recent publication reflects upon stakeholder engagement in participatory marine science projects in the EU ( Köpsel *et al.*, 2021). The authors describe coping strategies adopted by 30 projects and recommend seven practical actions to facilitate stakeholder engagement during the pandemic: “1) know your stakeholders (better than before), 2) strengthen existing relationships, 3) do not go 100% digital, 4) re-think your offline methods, 5) stay flexible and keep it simple, 6) apply lessons in post-pandemic engagement, and 7) account for the COVID-19 circumstances in your research results“ ( Köpsel *et al.*, 2021).

The CLIMAFRI project sought to reduce flood risks in Togo and Benin by integrating science-based data with insights from local stakeholders and communities. The project used virtual stakeholder workshops instead of the planned physical ones ( United Nations University, 2020). Community-based participatory research by Nguyen *et al*. (2020), in the context of HIV shifted the stakeholder-led steering committee meetings from in person to remote. The EU-funded project, ART-Forum, had planned interactive workshops with experts to simulate scenarios of autonomous driving ( Interreg, 2021). In addition, to facilitate dialogue and turn-taking, an online Delphi study was conducted with several iterative runs. Tobin *et al.* (2020) reflected on the methodology changes of their research on marine microplastics. They used three-hour online workshops instead of five-hour physical workshops on site. As an adaptation to remote work, they asked the participants to watch expert videos for preparation purposes ( Tobin *et al.*, 2020). Reflecting on their experiences, the authors recommended further research exploring the facilitation of online workshops, such as using breakout rooms.

Many projects relating to the effects of the COVID-19 pandemic on different aspects of life, relied on crowdsourcing to collect data. Crowdsourcing is defined by a type of participative online activity implemented by individuals or groups to collect data ( Estellés-Arolas & González-Ladrón-De-Guevara, 2012). For example, the open portal coronarchiv contains personal memories, e.g., diaries, photos, or social media chats reflecting life during the coronavirus pandemic ( Coronarchiv, 2020). In addition, the Corona Data Donation project has collected data like temperature and heart rate from over 500.000 volunteers’ wearable fitness devices Robert Koch-Institute, 2020).

Due to the novelty of the COVID-19 pandemic and the related disruptions for our lives, empirical knowledge about the changes for participatory research are still not well researched. The literature review showed that many participatory research projects already adapted their methods as an answer to the pandemic situation. However, a reflection about these changes is still lacking. The paper thus aims to answer to the research question how methods of participatory researhc can be adapted to face the challenges of the pandemic and which shortcomings and possible advantages for participatory projects are related to these changes. For this purpose, the paper reflects upon the European research project TRIPS.

### 1.2 Case study – The EU-funded project TRIPS

The project developed and applied a participatory research approach to increase the accessibility of public transport for persons with disabilities. TRIPS put forward a co-design approach that underpins Mandate 473: Design for All to eliminate discrimination and improve access to mobility services for all ( European Commission, 2020). The project developed and applied a participatory approach that aimed to 1) co-produce knowledge on existing barriers in transport, 2) co-create solutions for making transport more accessible, and 3) co-evaluate the resulting prototypes and services in the seven cities, i.e., Bologna, Brussels, Cagliari, Lisbon, Sofia, Stockholm, and Zagreb. The Co-design-for-All methodology creates the conditions for the equal participation of all citizens in open innovation and the development of inclusive mobility designs from their inception. In doing so, the project addressed the expected impacts of the call to help regional authorities and businesses design digital transport solutions that cater to individual needs.

The mission of the TRIPS project was to develop and prove the social value and validity of a co-design-for-all methodology that enables equal access to open innovation to all citizens, including those with disability. To this end, seven pilot case studies were planned that demonstrated the value of the approach and provided reference examples by applying it in seven European cities.

Consequently, achieving genuine participation and hands-on involvement was paramount for the project. Hence, attention to achieving and maintaining this focus, despite the complications presented by COVID-19, is a top priority for the project.

**Figure 1.**
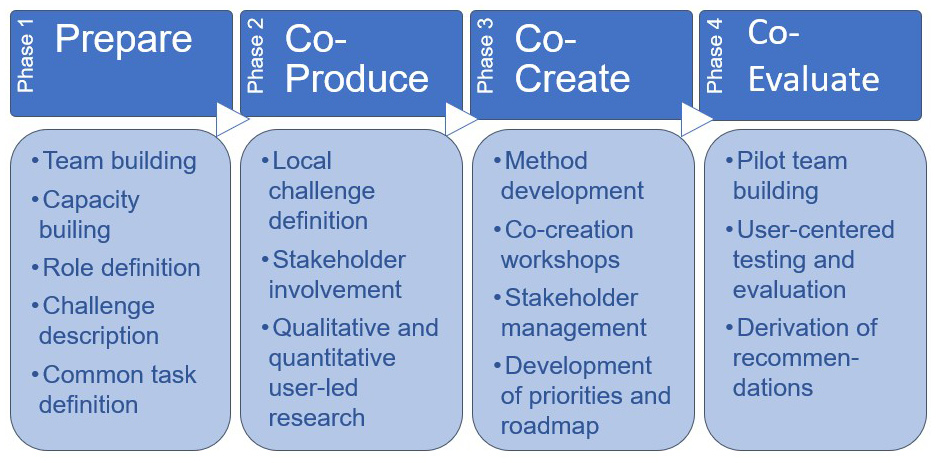
Original methodology of the TRIPS project.

The project started in February 2020 and will end in January 2023. The first two phases are finished (see Figure 1). Phase 3 (“co-create”) is ongoing and will be complete in spring 2022.

## 2. Reflection on participatory TRIPS methodology

### 2.1 Initial TRIPS methodology based on the project description

TRIPS is based on participatory case study research ( Reilly, 2010). It actively involved working groups of users and representatives of the transport community in all phases of the research and innovation process, from conceptualising the study to report writing and dissemination. It is ideologically oriented and emancipation-motivated, proposing radical changes in the social processes and innovation structures that shift the balance of power in knowledge production and use for understanding and responding to users’ mobility needs. TRIPS seeks to emancipate the users to play a central role throughout the innovation process, from user research to prototyping, to business case development. Ten project partners work with disabled users and transport experts in seven European cities (Bologna, Brussels, Cagliari, Lisbon, Sofia, Stockholm, and Zagreb). The project will demonstrate how people with disabilities can play a central role in designing inclusive digital mobility solutions when empowered.

As shown in Figure 1, the methodology of TRIPS consists of four phases: 1) preparation, 2) co-definition, 3) co-creation, and 4) co-evaluation. The methodological approach of TRIPS drew together diverse methods from a broad range of academic fields to support open, collaborative innovation that engages users and communities ( Baldwin & von Hippel, 2011). The project extended Wright and McCarthy’s (2015) notion of participatory design which stated that ‘knowing the users” in their day-to-day lives involves understanding what it feels like to be that person and their situation from their perspective. First, the project identified the gaps between user needs and preferences regarding existing urban transport, future mobility trends, and institutional and cultural barriers that prevent institutional actors from meeting those needs (see Qualitative Insights report here).

### 2.2 Initial challenges of disabled persons participating in transport research

Although participatory research is related to various benefits for the research process and the outcomes (c.f. Jagosh *et al.*, 2012), multiple challenges also affect it (c.f. Cargo & Mercer, 2008). However, a comprehensive overview of challenges that apply specifically to participatory research projects that involve persons with disabilities is still lacking. The TRIPS consortium anticipated various challenges at proposal stage. We clustered these around five key themes:

- **Challenges of spatial accessibility**: to find accessible premises for regular meetings of working groups that are easy to reach for everyone
- **Challenges concerning digital divide**: to agree on a common communication channel, e.g., emails, among the members of the working groups, can be challenging due to the different access requirements and needs
- **Challenge of balancing individual and common interests**: to find a balance between the interest of individuals and the group as the involved groups of persons with disabilities are very heterogeneous in terms of impairments and needs
- **Challenges about role ambiguity**: to facilitate a shared understanding and agreement of roles with disabled people as the experts for their lives, yet considering that the project team does not know everything related to specific impairments
- **Challenges with scheduling**: to coordinate meting dates and times that can accommodate the daily routines of persons with disabilities and their carers and the busy working schedules and family commitments of transport experts and other institutional stakeholders.

### 2.3 Methodology adjustments as a reaction to the COVID-19 pandemic

One of the main aims of Participatory Design (PD) is the active involvement of all stakeholders as co-designers ( Holone & Herstad, 2013). Participation can be invited but cannot be imposed as a one-size-fits-all approach ( Thiollent, 2011). Participants constantly negotiate it to become relevant to their current situation in a meaningful and culturally appropriate way. The COVID-19 pandemic is a situation that demands negotiation of participatory research practices beyond physical proximity to maintain the ethos of participation.

The following section presents the aspects of the COVID situation which motivated changes of the methods employed to maintain the ethos of the initially intended study. The changes of the methods were mainly initiated by the scientists of the project team at the beginning oft he project. However, some of the adaptations of the methods were also initiated by the local working groups during the process, such as the selection of co-creation methods. Table 1 summarises the objectives of the TRIPS project and compares the original methodologies to the adapted methods that were finally used. As shown here, we drew on the personal strategies employed by people with disabilities to stay in touch remotely, be socially connected while physically distant, and pay special attention to methods where absence and delays can be considered qualities rather than problems. The table is followed by elaboration of details.

**Table 1.** Comparison of the original and alternative methodologies.

Objective	Originally planned methodology	Alternative or adjusted methodology
To empower disabled citizens to participate in research and development and facilitate the research amongst peers ^1^, their access needs, mobility requirements, and attitudes towards future mobility solutions.	Establishment of the user community and working groups, consisting of users with disabilities, transport providers, city authorities, assistive technology suppliers and other interested parties. Training of the working groups for empowering them to apply methods.	The established working groups in seven project cities held remote meetings and were trained by the project team by using virtual meetings.
To identify barriers that persons with disabilities face before, during, and after their travel with public transport	Shadowing of public transport users during their trips and subsequent questioning.	Social media content analysis performed by local user leads.
To acquire in-depth information and insights concerning the travel patterns, attitudes and opinions of people with disabilities	Face-to-face interviews by researchers	Online peer-to-peer interviews
To gauge people's attitudes towards future mobility systems	Online survey to be disseminated during conferences and workshops following a multimedia presentation of the mobility systems.	Online survey without the audio- visual presentation and support. Recruiting survey participants relied solely on word of mouth and extensive communication.
To develop a multi-dimensional metric to measure the accessibility of different public transport systems regarding travel needs, such as timing, comfort, feeling of security of people with disabilities	Focus group workshops to be held online or face to face (originally not defined) involving stakeholders' representatives	Focus group workshops implemented online and supplemented by an online survey.
To review mobility solutions together with stakeholders and co-develop design concepts for future mobility solutions that are equally accessible, intuitive, and friendly to all users.	In-situ innovation workshops in Brussels	Online interactive co-design workshops of people with disabilities delivered in their native languages, for the seven cities.
To discuss the institutional barriers to the appropriation and implementation of suggested technologies and discuss potential solutions.	In-situ workshops in seven cities with local stakeholders	Online interactive co-production workshops as flexible units with stakeholders delivered in native languages for the seven cities.
To co-create collaborative methods with the seven groups of persons with disabilities working in the project.	A string of in-person activities allowed the methodological approach to be designed in short bursts of engagement.	A long series of regular 1:1 sessions which used a combination of qualitative research methods.
To deploy the collaborative methods as peer-to-peer activities in each of the seven cities in the project.	Peer-to-peer in-person activities within the whole group or only parts of it	Peer-to-peer online activities with one to three whole-group workshops
To generate knowledge, ideas, and concepts for improving the mobility of people with disabilities through collaborations between people, technology, and society.	Research through design by different methods like workshopping and sketching	Use of predesigned kits, including artifacts, maps, or photographs, to facilitate remote co-creation and creatively share concerns and knowledge using virtual ways of sketching.

The first objective of TRIPS is to empower citizens with disabilities to participate in the research over the entire project. Initially, it was planned to establish working groups in each of the seven partner cities, consisting of 10 to 15 people with different access needs and local representatives, like transport providers. The local working groups were composed of a local user lead (LUL), who are disability activists; the core user team (CUT) comprising mostly people with disabilities themselves and institutional stakeholders, representatives of transport organisations. Typically, the LUL role and core user team members comprise people with different impairments (e.g., wheelchair users, visually impaired individuals, hearing impaired individuals, short stature persons, etc.). In partner cities, the LULsare responsible for contacting potential members of the CUT and, with the local coordinators' support, who is a scientist from the project consortium. The LUL were contacted by the project member European Network of Independent Living (ENIL) and in some cities were members of the network. The LUL and CUT were trained by the project team to conduct the methods, like for example performing an interview study. The training is described in ( Vasconcelos *et al.*, 2021).

Another project objective is to identify barriers persons with disabilities face when traveling on public transport. Initially, TRIPS intended to conduct a shadowing study in the seven partner cities to observe users taking public transport during their trips; understand their end-to-end journey challenges, and explore the criteria affecting their transport-related decisions. Unfortunately, given the partially discontinued public transport service when the shadowing study should have taken place and the high risk of infections when using public transport, the shadowing study could not be conducted. Instead, the team pursued a social media content analysis to retrospectively identify barriers to public transport use. Social media content analysis utilises user-generated social media data as a barometer for attitudes regarding specific topics ( Lai & To, 2015). In the context of TRIPS, the social media content analysis provided insights into the discussion regarding public transport use in each city.

Another TRIPS objective is to acquire in-depth information and insights concerning the travel patterns, attitudes, and opinions of people with disabilities. For this purpose, a qualitative interview study is initially planned to be conducted face-to-face. Instead, the interview study was conducted online using videoconference systems due to the social distancing measures. Each LUL interviewed seven persons from their cities in their native tongue. See Alčiauskaitė *et al.* (2020) for more information.

A survey sought to gauge people’s attitudes towards future mobility systems. The survey was conducted online as planned; however, its dissemination approach shifted to using conferences, workshops, and webinars to present the mobility systems vividly. The consortium had originally planned an online questionnaire to collect data via online voting during conferences and workshops organised around punctuated events, such as the International Disability Day on December 3, and via TRIPS working group members during their interactions with other disabled citizens during local meetings. The idea was for partners and working group members to participate in various local events, present a multimedia presentation of the mobility systems allowing users to ask questions to understand the mobility concept presented, and then answer several questions regarding their intention to use it, the value of such systems and possible ways they would adapt it to fit their lifestyle better. Unfortunately, the survey was conducted without audio-visual presentation and support due to on-site conferences and workshops being cancelled. Therefore, the recruitment of participants had to rely solely on word of mouth and extensive dissemination ( Alčiauskaitė *et al.*, 2020).

One of the main objectives of TRIPS is to develop a multi-dimensional metric to measure the accessibility of different public transport systems, the so-called Mobility Divide Index (MDI, Bagnasco *et al.*, 2021). To determine the index structure, investigate and prioritize the main dimensions that could influence people with disabilities’ travel experience, two workshops were planned and organized online, recruiting the TRIPS local working groups. In addition, an online survey, not initially anticipated, was conducted to assess and weight the identified dimensions of the MDI from a user perspective (N = 113).

In-situ workshops in Brussels were planned to co-create concepts of future mobility solutions that help overcome existing barriers. Instead, the consortium conducted online, interactive, co-design workshops with people with disabilities in their native languages in seven cities ( Hoogerwerf *et al.*, 2021).

Five workshops with stakeholders were initially planned in Brussels over two days adjacent to a yearly accessibility conference. The aim is to discuss the institutional barriers to the suggested solutions and identify facilitators and barriers to their implementation. However, the capabilities of local teams and their facilitators varied. In some cases, users' level of impairments dictated their endurance in holding long meetings. Hence, we adapted the final research design to meet their needs. As a result, we planned the workshops in modules that facilitators could combine to adjust the length of each workshop. As a result, we held, organised, and coordinated more workshops. In particular, we conducted thirteen online workshops with 100 participants over two months ( Hoogerwerf *et al.*, 2021).

TRIPS also sought to devise a co-design methodology for all, with accessibility principles of engagement and a strong stance on access, participation, and ownership. The methodological foundations TRIPS built upon were the physical presence and face-to-face modes of inquiry, yet, people with disabilities have always faced additional barriers to physical mobility that would allow for this to happen (e.g., Wilson, 2003). COVID-19 also challenged access to public transport for full participation in society and independent living to the fore as TRIPS tried to maintain its intended spirit and participatory ethos.

The co-design methodology development was conducted entirely via participatory online workshops and iterative, action research. It encompasses collaborative planning and execution of tasks, then reflection sessions for critical reflection and adjustment. This work started with defining a theoretical foundation for participatory inquiry in the context of the current limitations imposed by the pandemic. This established the stage for what constitutes the primary ongoing process of the TRIPS methodology: to co-create collaborative methods with the seven CUTs of persons with disabilities working in the project. During this process, each LUL and LC created localized versions of the methods online and deployed peer-to-peer online activities in each city. Deployment took place during March to May 2021 in each city. Workgroups engaged in a series of activities to formalize their unique identity and their vision for what they wanted to achieve within the duration of TRIPS.

To make up for the loss of in-person activities, we engaged each group in a string of conversations to anchor the methodologies into strongly-held local concerns and to guarantee that the processes remained within our understanding of co-design and co-production, despite the apparent limitations of online work. This work unfolded as a long series of regular one-on-one sessions. We used a combination of qualitative research methods: semi-structured interviews, open-ended activities, writing exercises, surveys, offline activities, etc. Our focus was to create a dynamic working rhythm and generating mechanisms to allow for heterogeneous interests and in-depth understandings to come forward. From October 2020 to May 2021, each city was involved in 10 to 16 one-to-one sessions, one to three whole group workshops, two to four offline activities. We regularly had two to four participants in the one-to-one sessions, and the workshops were open to the entirely local team (CUT) in each city. Although the number of activities varied, as in this work, we recognize that not all cities arrive at this process on the same footing. Their needs, preferences, and challenges are unique and contingent on their local contexts and therefore require ways of working that emerge from within each of the groups involved. Specifically, some cities require more regular meetings with the project team and closer monitoring, whereas others practice a more independent working style.

**Figure 2.**
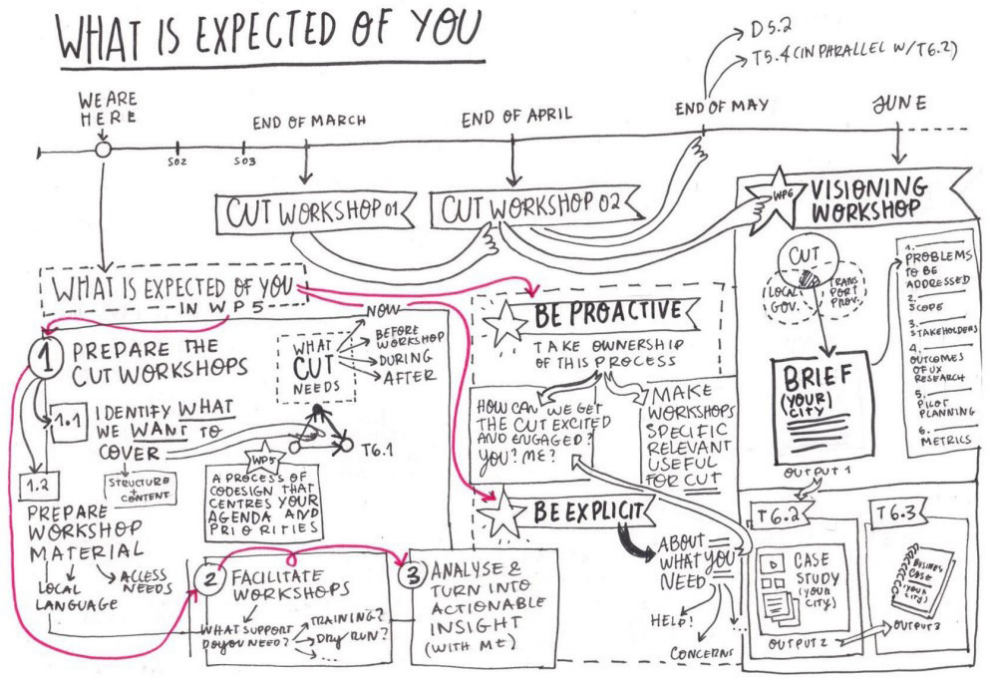
Exemplary sketch used in a workshop.

This approach allowed us to tailor each interaction to local and personal preferences. Of course, not everyone had the same experience, but we worked towards shared understandings and convergence through various interactions and strategies. In practice, this work was done using the following techniques:

- **Workshopping**: We aimed to create an experience where individual narratives coexist with complex understandings of collective knowledge, leading to a great diversity in outcomes.
- **Brainstorming**: Brainstorming allows for a broad range of knowledge to manifest, be shared, and co-created. This has a dual effect on user involvement: it generates possibilities and equally improves the social dynamics of exchange as a basis for shared meaning.
- **Sketching**: Through sketching, we aimed to explore notions of collaborative visual thinking, in which non-verbal techniques like drawing are used to represent unified action ( Figure 2). Live and online sketching was performed by a professional designer to visualize the work progress and record results.
- **Interviews**: Interviews elicit individual knowledge and narratives. We sought to use them as open engagements where personal stories guide participants and interviewers in the narration of lived experience.

## 3. Discussion

### 3.1 Reflection on the participatory methodology adaptation in TRIPS

The applied method's effectiveness, feasibility, and goal-reaching capacity are reflected upon below. In doing so, the pros and cons of each method are discussed.

***Empowerment of citizens and building of working groups.*** In the TRIPS project, users with disabilities take a central role in the co-design process, not only as passive observers and reviewers, but as the individuals initiating the changes and proposals for future mobility solutions. However, building and maintaining relationships remotely has been a very challenging task. The COVID-19 pandemic-adjusted methodology required strong leadership skills from all LULs. They recruited and established the local working groups and implemented the co-design in their city. They all reported facing the following challenges: (a) not all people with disabilities are comfortable using digital technologies; (b) approaching transport providers or city authorities online was complicated; (c) virtual meetings seemed less attractive than offline ones. On the other hand, virtual meetings allowed them to be more flexible with arranging the meetings, making them easily accessible to more people and reducing logistical effort and cost. It should be emphasized that the training of disabled citizens as co-researchers was facilitated by online formats, because the recording of training sessions, e.g., how to use a digital sharepoint and teamsites, allowed them to study the material in their own pace after the training. Furthermore, content was translated and subtitled live with the help of programs, which increased accessibility for specific groups of sensory-impaired people. In addition, having more frequent online meetings than planned for physical meetings, helped to face the challenge to create and maintain interpersonal relationships as empasized in the literature review by Hall *et al.* (2021).

***Peer-to-peer interviews.*** Conducting the interview study online using a peer-to-peer approach had various advantages and disadvantages. One of the prevailing advantages was the familiarity and intimacy created by the peer-to-peer interview setting, as reported in earlier studies in facilitating critical inquiry ( Peck *et al.*, 2021; Schmidt, 2017). Presumably, the familiar and trustful atmosphere enabled a greater openness from interview partners and correspondingly more in-depth information. Conducting the interviews in the native language was another advantage of the method changes. Using online and distance formats increased accessibility of specific groups of people with disabilities with severly restricted mobility or who are particulary afraid of an infection with the coronavirus. The cons of online interview studies are sampling issues due to the level of access to and competency in using digital systems ( Duffy *et al.*, 2005). These negatively impacted the TRIPS‘ interview study. It was difficult for LULs to identify, select and recruit disabled interviewers with access to and sufficient competency in using digital tools, like videoconference programs.

***Social media content analysis.*** As outlined in other studies regarding participatory approaches in times of COVID-19, the use and analysis of secondary data, such as media content, may be an appropiate alternative for fieldwork ( Adom *et al.*, 2020; Jowett, 2020). The social media content analysis revealed itself as a method that produced many insights into the thoughts and attitudes of persons with disabilities regarding their daily mobility challenges (see Alčiauskaitė *et al.*, 2020). However, conducting the study was somehow challenging, as the method was new to the project team and the LULs conducting the research. Detailed instructions in the form of a step-to-step manual were essential for guiding the procedure and were thus developed by the project team. Some of the LULs who conducted the social media search faced difficulties reaching the minimum number of 30 social media entries, whereas others achieved the limit more rapidly. This implies that in addition to the number of social media posts per city, the ability of people to search online, access the websites, and use appropriate search terms also differed. Furthermore, not all LULs were familiar with social media and had access to all relevant platforms. Thus, accounts were created to enter the social media platforms. Another challenge arises when reflecting the method in terms of inclusiveness for specific groups of disabled users. In detail, it must be assumed that some groups of disabled users, among them especially people with cognitive and intellectual impairments, are not fully represented in social media content analysis, because they are less likely to have access to the internet and thus social media ( Chadwick *et al.*, 2013). To conclude, the social media content analysis proved to be a valuable and feasible method to identify mobility barriers discussed in a specific geographic context. However, the social media content research quality strongly depended on the people's digital skills conducting the investigation. Thus, people conducting the study should be knowledgeable in/trained to use different social media platforms and select appropriate search terms. Furthermore, limitations with regard to the representativeness of specific groups of disabled users should be considered.

***Virtual co-design workshops.*** The adaptation of the co-design process to online activity required pilot co-design workshops to test the suitability of digital tools and the planned methodologies. Thus, a pilot workshop was conducted for each co-design session format (creating mobility solutions and identifying institutional barriers). The pilot workshops aimed to train the seven co-design workshop facilitators, who were often people with disabilities, mostly without many experiences in organizing and conducting workshops. After the training, the local facilitators conducted the workshops with participants mainly from their cities (Bologna, Brussels, Cagliari, Lisbon, Stockholm, Sofia, and Zagreb). The pilot training workshops were beneficial, as their evaluations revealed several recommendations for the workshops in the seven cities:

- The aims and purposes of the workshops should be clear from the start
- A guide for the local facilitators to explain how to deliver the workshop would be beneficial
- The material (hand-outs and worksheets) should be shared beforehand to allow the participants to familiarise themselves with the content
- Additional guidance for facilitators as footnotes would help in the delivery
- Pictures/figures should be verbally explained to increase accessibility for visually impaired users
- The content on the presentation slides should be reduced
- The time allocated should be adapted to allow more time for discussion of innovative concepts
- Relate the exercises to each other and the bigger picture and spend enough time to present the aim of the workshop
- If possible, during the exercises, display a timer counting down the available time
- During the exercises, repeat the question/task every two minutes or give an additional prompt
- Break-out rooms are essential for facilitating discussions in smaller groups
- Virtual warm-ups are necessary to replace face-to-face small talk
- After each exercise, allow time for participants to share their answers to make it more interactive
- Remind people periodically to state their name when starting to speak
- Remind speakers to speak slowly and clearly to facilitate live captioning for people with hearing impairments

As a result of the feedback in the pilot workshops, several actions were taken. For instance, all content was made accessible according to best practice guidelines, like the Accessible Online Event Toolkit of the European Disability Forum. Furthermore, the TRIPS consortium revised content to streamline running time and enhance productivity. In addition, a guide for facilitators was produced, alongside delivery notes in the slide deck to facilitate the local workshops. Furthermore, local facilitators paired up with a project team member, which helped them prepare the workshops and provided feedback and support.

Even though a pilot online training workshop was conducted in both workshop formats, the local workshops encountered several challenges. First, the importance of preparing the grounds for a shared understanding beforehand was emphasized. For example, the participants requested that mobility concepts be introduced in the briefing document to reflect upon them and facilitate creativity during the workshop. Second, facilitators asked for support to administer online tasks, such as sharing the PowerPoint presentation. Finally, participants mentioned that compared to *in-situ* workshops, the required level of interaction and focus was more intense. These were due to levels of eyestrain, discomfort, and attention to more detail. Therefore, expectations regarding concentration levels, physical endurance, and productivity should be adjusted for online settings in future research. Accordingly, enough breaks should be planned. These recommendations might help to overcome the challenge described by Woodward *et al.* (2020), that people with visual or hearing difficulties may struggle to engage fully in online discussions.

***Online survey.*** COVID-19 deprived us of the interpersonal interaction preceding the survey as all physical events, conferences and workshops were canceled; even some online events were postponed. The survey was thus disseminated using social media channels and specific groups for people with disabilities. To ensure that the survey was clearly understood, we designed the questionnaire in consultation with the leaders of the TRIPS working teams. We pilot-tested the translated versions with the members of our working groups.

***Online focus group workshops.*** The project LULs performed two online focus group workshops to achieve the project objective of investigating and prioritizing the main variables influencing persons with disabilities during their daily travels on public transport. These variables would constitute the core structure of our Mobility Divide Index (MDI).

Since the target audience was people with different impairments, we had to prepare the material in advance, using simple words and applying easy-read techniques to make presentations accessible for all, especially for people with reduced vision.

The size of focus groups is generally recommended to be between seven and ten participants. However, considering the topics' complexity and the heterogenity of disabilities of participants, after a preliminary introduction and discussion with all participants, we divided them into three focus groups (i.e., three concurrent breakout rooms) of three to four people. Each group was tasked with reflecting on a limited set of aspects of their daily journeys on public transport. This choice allowed us to manage the online sessions better and derive more insights. This recommendation is in line with the finding of Tobin *et al.* (2020) who recommended breakout rooms for smaller group discussions.

Since online focus group workshops can be conducted anytime and anywhere, sessions must have a limited duration to avoid participants getting bored and distracted. Therefore, we planned sessions of two hours each. We narrowed the discussion guide to a few key topics to respect the scheduled time slot. This influenced our study: while we carried out a deep investigation of the main issues that affect the mobility routines of people with disabilities, we did not thoroughly examine the prioritization of these issues. We took the opportunity to launch an online survey to collect views on the importance of different MDI variables for persons with disabilities agreed during online focus groups. We disseminated it online via ENIL channels. In addition, we contacted disability NGOs representing persons with disabilities. The latter required a significant commitment from the members of the respective organizations and resulted in a more extended data collection period to reach a substantial number of respondents.

***Software adaptations.*** Instead of meeting physically, videoconference systems were used to meet within the project team, the project team with the CUT and the seven CUT among themselves, in what became a longer one-to-one process for specifying and establishing specific local challenges and work methods. These online tools generated advantages, e.g., it was easier to stay in touch without traveling, which is challenging especially for disabled people, and disadvantages, e.g., it became clear that creating engaging activities was much more complicated, and the potential for misalignment was higher. In addition, these online interfaces came with their accessibility issues, which influenced the outcomes in part (e.g., group work and in-depth discussions were made more difficult in those circumstances) and forced us to work in much smaller groups.

To communicate the practical software setup, the groups needed to participate in an online session, and we, therefore, created an access needs protocol. This protocol was intended to be used for all project-related work and any other activities that a group is invited to attend, e.g., a meeting with the city council. To create this protocol, we followed Sandra Lange’s ‘Access Rider Exercise’, prompting each group to articulate what they would need, both individually and as a group, to engage in online activities ( Lange, 2020) fully. The access needs protocol was meant to be used for each group to create and occupy a shared online space while shaping their interaction conditions in that space.

Moving to a digital working space also required a greater need for establishing online collaborative working processes that catered for varying levels of digital skills of people with disabilities. Each city required different levels of support and tended to elicit unique working dynamics. This digital setup did not reflect how most groups involved in the project typically work, e.g., having a shared folder with up-to-date documents was a surprisingly challenging task. The impetus for having online documents has come not only as a means to produce deliverables (EC project reports) but also as the only way to document and share knowledge between the CUTs and the partners involved in the project. In other words, the TRIPS project worked digitally, but we need to pay continuous attention to guaranteeing that these online spaces are truly shared environments. To convice and train people with different access needs to use the same online work tools, like sharepoints, was challenging. Ultimately, creating efficient and productive online working methods in a multiple-partner project requires a significant amount of effort and ongoing attention.

To conclude, the reflection of the adaptions of the methodology due tot he COVID-19 pandemic, revealed several challenges for participatory research with people with disabilities.

### 3.2 Reflection on the initial challenges and the actual challenges faced in participatory research with persons with disabilities

During the proposal writing and planning of the TRIPS project, various considerations were identified to facilitate the participation of disabled persons in our collaborative processes. As a result of the adaptation of the methodology due to the pandemic situation, the challenges faced in the project changed.

The initially identified challenges of spatial accessibility were addressed by not meeting in person, rather meeting virtually. Finding a way to adapt the methods to online meetings facilitates the participation of people that would have otherwise not been able or hard to attend physical meetings. To make up for the loss of participatory workshops in person, TRIPS used a string of 1:1 conversations to anchor the methodologies into firmly held local concerns and guarantee that the processes remain within our understanding of co-design and co-production, despite the apparent limitations of online work.

Concerning the challenges of the digital divide, we found that actual challenges were higher than anticipated for the virtual participatory work. The required digital literacy for participation was higher than anticipated before because the physical meetings would not have required digital skills. In using digital tools for collaborative work, e.g., Google docs, the CUT find it difficult to learn how to use those tools. As more documents started emerging, we were also faced with the practical difficulties of creating and maintaining collaborative processes online - keeping documents in shared folders up to date was surprisingly hard to task and required ongoing upkeep and management. Furthermore, the video conference tools used allowed for live captioning and translating of the content, thus improving the accessibility of the meetings.

Regarding balancing individual and common interests, the project faced the same challenges when adjusting the methods. Thus, no statement can be made whether the adapted methods impacted this challenge.

With respect to role ambiguity, we observed some differences compared to the anticipated challenges. For example, the training of co-trainers that implemented the methods, like co-creation workshops in their local groups, was facilitated by the more frequently virtual meetings. Furthermore, our reflection supports the hypothesis of Marzi (2020) a lack of control due to using online formats and digital tools also provides a means to equalize power-relationships between researcher and disabled participant and thus ensure equity.

Online meetings were more manageable in some respects. We did not have to consider the accessibility of venues and traveling to them and could be more spontaneous in our planning. Coordinating meeting times that fit the different daily routines of disabled people and the carers, on the one hand, and the working and family obligations of transport experts on the other were more manageable. Conducting fieldwork as a long series of regular 1:1 sessions allowed us to capture heterogeneous interests and in-depth understandings, allowing deeper rapport with CUT members. Closer collaboration between the CUT and the project team was possible because traveling was no mandatory requirement to meet.

Of course, conducting participatory activities online brought unique and unanticipated challenges and created other conducive forms of involving disabled people. Whereas in some cities, we found that the groups could adapt the imposition to work online to their advantage, we also observed the setbacks in aligning and keeping motivation up. In hindsight, using a combination of online and offline methods can create added value and facilitate participatory research. Although, we put this forward speculatively informed by the current experiences of running hybrid online-offline programmes and the unique challenges they bring with them.

### 3.3 Lessons learned and recommendations

Several lessons can be derived from the assessment of the applied methods. The following eight practical recommendations guide future participatory research projects that face challenges in conducting research *in-situ*.

**The need for piloting new methodologies**: Have a pilot of online workshops with “real” participants to facilitate the adoption of the method. We found it was worth the time. By piloting the online workshops, we identified several pitfalls, e.g., non-accessible technologies, that were then adressed before the actual workshops. By engaging disabled users in the online planning and preparation meetings is helpful for adapting the methods to the specific needs of people. Also, piloting and training sessions should be recorded to allow disabled users, who are trained to conduct the workshops later on, to study the material in their own pace afterwards.

**Absence as a feature**: Try to value the other side of the impeded cooperation on-site. There are also potential advantages to distance and online formats, such as increased time for reflection, broader participation, and improved attention due to the sharing of documents and the joint and simultaneous processing of documents and tasks. Working with shared online documents facilitates disabled project partners to work at documents and tasks in their own pace.

**Mixed presence**: Be open to experimenting with new ways of being together at a distance. Mixed presence, which combines distributed and collocated collaboration, might create meaningful exchanges when prepared carefully. Mixed presence might also facilitate opportunities to empower people with disabilities through choosing appropiate formats for knowledge and capacity building.

**Personalised and localised:** These new ways of working together will allow us to tailor each interaction to local and personal preferences and specific circumstances, like local equality regulations. This also means that a shared understanding and convergence through various interactions and strategies must be facilitated. Adapted online methods allow for more frequent contact and thus a closer cooperation between researchers and disabled people as well as disabled people among each other.

**Advantages and disadvantages for equity issues**: On the one hand, virtual participatory methods such as online workshops expand the reach of the research and thus facilitate the participation of vulnerable-to-exclusion citizens who would otherwise have not participated in *in-situ* workshops, like people living in the suburbs or rural areas or people challenged to leave their homes without the help of others. On the other hand, virtual implementation of participatory methods excludes other groups, such as persons with low digital literacy. It should be considered that by using online formats some groups of disabled users, such as people with severe cognitive or mental impairments are mostly excluded.

**Stay connected:** It is important to stay connected with the participants after virtual workshops for further inquiries. Virtual communication methods, such as e-mails, can be used to ask participants to evaluate the method and additional suggestions. Thereby, follow-ups/subsequent ideas can be included in the process and continuous participation facilitated. One-to-one online meetings can help to increase motivation of honorary project partners in the research process and to adapt the methods and formats to the individuals‘ needs.

**Online methods require more focus as they are highly demanding for participants’ attention:** Conducting creative workshops online instead of on-site requires more focus and smaller-scale formats, like meetings and workshops, as maintaining attention in online settings is challenging. Thus, people with disabilities should not be overloaded with information in online formats. It is thus recommended to plan more but shorter and less dense online workshops.

**Stay flexible**: Participatory approaches are inherently flexible and need continuous adaptations. Beyond that, ever-evolving situations, like a pandemic, require dynamic adaptations of research scopes and methodologies, as also recommended before fort he reflection of stakeholder engagement during COVID-19 by Köpsel *et al.* (2021) Maintaining flexibility while accepting limitations can ensure the quality of the process. In this way, the specific requirements of people with disabilities can be considered that might not be known from the start of the project.

To summarize, the assessment of the methodological changes and adaptions during the TRIPS project showed us that there are many opportunities to deal with challenging situations. Therefore, we should not only perceive disadvantages but also value opportunities.

### 3.4 Next steps and further work

The reflection on the methodological adaptations for the TRIPS projects as a reaction to the COVID-19 pandemic leaves several unanswered research questions that need to be further considered in research and practice. First, that participatory approaches rely on trust, continuous rapport, and exchange, an emerging research question deals with the issue of facilitating trust-building, and a collaborative working spirit and productive atmosphere in the light of social distancing. What are possible ways to foster a trustworthy working atmosphere with digital and non-digital methods? How do we replace valuable small talk and networking during workshop breaks of workshops and events when they are conducted online? How do we give local teams a sense of ownership? How can there be a structure that nurtures ownership and governance of working teams?

Further research should also address how virtual methods should be adjusted to the needs of different users. Furthermore, from the perspective of inclusivity, it seems worthwhile to find ways to implement easy-to-read material in virtual conferences and workshops.

Before the pandemic, projects were implementing participatory research methods for remote work when it was difficult or impossible to work with participants in a co-located context. Exemplary methods are cultural probes ( Gaver *et al.,* 1999). These methods should be considered in light of the COVID-19 pandemic.

The TRIPS project will continue to implement methods of participatory research. The next step of the project will be the engagement of local users and institutional actors in 1) co-creating prototypes of future mobility solutions, 2) organising user testing of the prototypes and evaluation of the co-creation process, 3) developing the prototypes into local pilot demonstrators, 4) organising local user testing of local pilot demonstrators, 5) conducting business analysis of the local transport ecosystem and 6) developing the business case for the full-scale deployment of the local pilot demonstrators.The project will also enagge people with disabilities, public transport operators, and institutional actors in developing and validating policy recommendations, research priorities, and an industry roadmap for the mobility sector. Specifically, the TRIPS project consortium will validate the described design concepts and preferences as well as the derived policy recommendations, industry roadmap and research priorities with users and institutional actors from the seven cities.

## 4. Conclusions

The reflections on the adaptations of the participatory research methodology for the TRIPS project will help researchers and scholars to be more prepared for conducting participatory research during the ongoing and future pandemic. Keeping in mind that the adjustments caused by COVID-19-related restrictions are going to persist and be used to some extent in the future (e.g., more people are working from home, online workshops replacing face-to-face workshops, Thombre & Agarwal, 2021), researchers need to adapt their research design to the existing situation and turn the limitations into opportunities. This paper contributes to a better understanding of the functioning of remote participation and which of its aspects could be implemented in the future. Moreover, reflecting on the methodology changes showed that opportunities to deal with the new situation are manifold, and participatory research should not consider disadvantages but value the derived options. The reflection oft he case study showed ways for online formats to help „[…] to move beyond the rhetoric of “participation” toward more meaningful, holistic inclusion of people with disabilities into research and design“ ( Monteleone, 2018, p. 137) To conclude, the methodology reflection for the case study of the TRIPS project provides lessons learned from implementing research during the COVID-19 pandemic but also for other possible future changes and challenges.

## Consent

Written informed consent for publication of the participants details and/or their images was obtained from the participants//parents/guardian/relative of the participant

## Data availability

### Underlying data

No data are associated with this article.

### Extended data

Zenodo: Research material TRIPS, https://doi.org/10.5281/zenodo.5752461 ( König, 2021)

This project contains the following extended data:

- Guideline_briefing document_ media content analysis.docx (Guidelines for conducting the social media content analysis for the CUT, comprising a detailed description of the steps to perform the task)
- Interview guidelines.docx (Guidelines for conducting the peer-to-peer interview study containing semi-structured interview questions and instructions for the interviewer)
- TRIPS Workshop Design.docx (Short description of the workshop design for the co-design workshops)
- TRIPS_ Guidance document for Co-Design Workshops.docx (Guidance document for the co-design workshops, including detailed instructions for the recruiting of participants, structure, and tasks of the workshop, accessibility requirements, and technical instructions)

Data are available under the terms of the Creative Commons Attribution 4.0 International license (CC-BY 4.0).

## Footnotes

^1^ With the term ‚peers‘ people that also face disabilities are mentioned but who are not members of the project.
