# Supplementary material for: A reflection on participatory research methodologies in the light of the COVID-19 – lessons learnt from the European Research Project TRIPS
Source: Open Res Eur. 2022 Mar 29;1:153. Originally published 2021 Dec 16. [Version 2] doi: 10.12688/openreseurope.14315.2 (PMC10446030; doi:10.12688/openreseurope.14315.2)
Supplement: Peer review history

# Peer review: Reviewer response for version 2

**DOI:** 10.21956/openreseurope.15799.r28959

**Published:** 2023-08-18

**Authors:** Angelos J. Amditis, Christina Anagnostopoulou

**Affiliations:** 1 Institute of Communication and Computer Systems (ICCS), National Technical University of Athens, Athens, Greece

The authors adequately addressed our comments and recommendations, and revised the manuscript accordingly. Along with the changes according to the other reviewer's comments, the article's quality has improved and the work of the authors is presented in a more pertaining way.

Is the case presented with sufficient detail to be useful for teaching or other practitioners?

Yes

Is the work clearly and accurately presented and does it cite the current literature?

Yes

If applicable, is the statistical analysis and its interpretation appropriate?

Yes

Are all the source data underlying the results available to ensure full reproducibility?

No source data required

Are the conclusions drawn adequately supported by the results?

Yes

Is the background of the case’s history and progression described in sufficient detail?

Yes

Reviewer Expertise:

Intelligent Transport Systems, electromobility, smart mobility concepts, MaaS, autonomous, connected and integrated mobility, CCAM, C-ITS,  5G applications for mobility, logistics, citizen observatories, etc.

We confirm that we have read this submission and believe that we have an appropriate level of expertise to confirm that it is of an acceptable scientific standard.

# Peer review: Reviewer response for version 2

**DOI:** 10.21956/openreseurope.15799.r28958

**Published:** 2022-04-05

**Authors:** Vera Köpsel

**Affiliations:** 1 Institute for Marine Ecosystem and Fishery Science (IMF), University of Hamburg, Hamburg, Germany

Dear Authors,

 I was delighted to see how thoroughly you responded to my review comments and would like to see the manuscript published in its updated version.

 Personally, I am especially happy to learn more about the challenge of working with disabled stakeholders. 

 Warm regards

 Vera Köpsel

Is the case presented with sufficient detail to be useful for teaching or other practitioners?

Partly

Is the work clearly and accurately presented and does it cite the current literature?

Yes

If applicable, is the statistical analysis and its interpretation appropriate?

Yes

Are all the source data underlying the results available to ensure full reproducibility?

Yes

Are the conclusions drawn adequately supported by the results?

Yes

Is the background of the case’s history and progression described in sufficient detail?

Yes

Reviewer Expertise:

Stakeholder engagement in EU projects and transdisciplinary research; coordination of stakeholder engagement; online and offline engagement methods; challenges of stakeholder engagement during COVID-19; human geography; social scientific methods

I confirm that I have read this submission and believe that I have an appropriate level of expertise to confirm that it is of an acceptable scientific standard.

# Peer review: Reviewer response for version 1

**DOI:** 10.21956/openreseurope.15444.r28194

**Published:** 2022-01-20

**Authors:** Angelos J. Amditis, Christina Anagnostopoulou

**Affiliations:** 1 Institute of Communication and Computer Systems (ICCS), National Technical University of Athens, Athens, Greece

The article's first section provides a good summary of the challenges for participatory research in relation to the COVID-19 pandemic, and several adaptations as were decided by several projects. The presentation of TRIPS is well described in terms of overall project objectives and goals, having the aim to develop and apply a participatory approach that aimed to increase the accessibility of public transport for persons with disabilities, by 1) co-produce knowledge on existing barriers in transport, 2) co-create solutions for making transport more accessible, and 3) co-evaluate the resulting prototypes and services in seven cities. Literature review and sources are extensive and sufficiently provided to cover the information presented.

 First of all, I find the article lacking in terms of well defining the overall challenges of the endeavor that has to do with accessing, collaborating, and retrieving knowledge from people with disabilities from different countries that have different regulations and levels of penetration for smart or new mobility concepts. So all in all, a different reality for accessibility in transport. Thus, despite the extra restrictions caused by the pandemic, I could see similar issues being faced in the originally planned methodology. So in the end, these are not matters that are deriving necessarily from the conditions of the pandemic, but rather from the core of the project target that is to involve people with several disabilities and backgrounds. For example, the change from one f2f workshop in Brussels to several virtual co-design workshops worked positively to the quality of the results and facilitated the process in the end, although yes some people were not familiar with digital means or pilot workshops were needed to train the local organizers. I would imagine organizing one physical workshop instead would have neither provided a comfortable situation for disabled people nor would it have made it any easier for the work of the researchers themselves to communicate the message. So even results in a less enabling situation.

 The authors do not specifically explain what were the risks or challenges that they had predicted in their original methodology. Since from the start the target group of the study is disabled people and this factor itself introduces challenges on its own. The pandemic has driven the change to the original methodology and led to shifting a lot of activities online/remotely or even changing the activity itself. Although the modified new methodology added extra challenges, it seemed in some cases that it acted positively and succeeded in solving some issues – that possibly could have been there from the beginning. Thus, in the end, it could be argued that for said target group, specific already designed approaches when shifted to online surveys or virtual workshops with given guidelines and explanatory framework, can really create added value and facilitate research. This could result in recommending such approaches and even having them adopted as effective methods for participatory research or used complementarily for the target group, regardless of cases that impose restrictions. This comparison between the challenges faced when targeting disabled people against the challenges faced when targeting disabled people with the restrictions imposed by the pandemic is not clear, and this is a shortcoming. The comparison could have shown that despite this change being imposed by the pandemic in the end, the new method can help anyway and create better terms for such work even without imposed restrictions. The argument is that it seemed in their modified approach that the remote or virtual activities created a better environment for the group, contradicting their original plan.

 This would be a distinction that would make the recommendations of this work really valuable to the reader, while now the overall feeling for the lessons learned and recommendations part remain generic and not so impactful.

 I would suggest to generally edit the paper in order to use less repetitive and generic language throughout the sections - be more targeted in the descriptions - retouch upon the syntax and grammar, highlight what are the initial challenges and risks in participatory research targeting disabled people and transport, how are these issues being changed by the restrictions imposed for meeting in person, and how the new approaches and the lessons learned while enforcing them enhanced the work or even provided better results and should anyhow be considered for participatory research methods when dealing with disabled people.

Is the case presented with sufficient detail to be useful for teaching or other practitioners?

Yes

Is the work clearly and accurately presented and does it cite the current literature?

Yes

If applicable, is the statistical analysis and its interpretation appropriate?

Yes

Are all the source data underlying the results available to ensure full reproducibility?

No source data required

Are the conclusions drawn adequately supported by the results?

Yes

Is the background of the case’s history and progression described in sufficient detail?

Yes

Reviewer Expertise:

Intelligent Transport Systems, electromobility, smart mobility concepts, MaaS, autonomous, connected and integrated mobility, CCAM, C-ITS,  5G applications for mobility, logistics, citizen observatories, etc.

We confirm that we have read this submission and believe that we have an appropriate level of expertise to confirm that it is of an acceptable scientific standard, however we have significant reservations, as outlined above.

# Response

**Published:** 2022-03-14

**Authors:** Alexandra König

**Affiliations:** German Aerospace Center (DLR), Germany

*The article's first section provides a good summary of the challenges for participatory research in relation to the COVID-19 pandemic, and several adaptations as were decided by several projects. The presentation of TRIPS is well described in terms of overall project objectives and goals, having the aim to develop and apply a participatory approach that aimed to increase the accessibility of public transport for persons with disabilities, by 1) co-produce knowledge on existing barriers in transport, 2) co-create solutions for making transport more accessible, and 3) co-evaluate the resulting prototypes and services in seven cities. Literature review and sources are extensive and sufficiently provided to cover the information presented.*

*First of all, I find the article lacking in terms of well defining the overall challenges of the endeavor that has to do with accessing, collaborating, and retrieving knowledge from people with disabilities from different countries that have different regulations and levels of penetration for smart or new mobility concepts. So all in all, a different reality for accessibility in transport. Thus, despite the extra restrictions caused by the pandemic, I could see similar issues being faced in the originally planned methodology. So in the end, these are not matters that are deriving necessarily from the conditions of the pandemic, but rather from the core of the project target that is to involve people with several disabilities and backgrounds. For example, the change from one f2f workshop in Brussels to several virtual co-design workshops worked positively to the quality of the results and facilitated the process in the end, although yes some people were not familiar with digital means or pilot workshops were needed to train the local organizers. I would imagine organizing one physical workshop instead would have neither provided a comfortable situation for disabled people nor would it have made it any easier for the work of the researchers themselves to communicate the message. So even results in a less enabling situation.*

 We appreciate your comment on the differentiation between the general challenges of participatory research in the context of transport and the additional challenges due to the coronavirus pandemic and the related adaptations of methods. We added a section to this topic at the beginning of the manuscript. We tried to clarify that the aim of the paper is to show how the pandemic situation affected the methods and results of the participatory research. Thus, a more general reflection on participatory transport research is not in the scope of the paper.

*The authors do not specifically explain what were the risks or challenges that they had predicted in their original methodology. Since from the start the target group of the study is disabled people and this factor itself introduces challenges on its own. The pandemic has driven the change to the original methodology and led to shifting a lot of activities online/remotely or even changing the activity itself. Although the modified new methodology added extra challenges, it seemed in some cases that it acted positively and succeeded in solving some issues – that possibly could have been there from the beginning. Thus, in the end, it could be argued that for said target group, specific already designed approaches when shifted to online surveys or virtual workshops with given guidelines and explanatory framework, can really create added value and facilitate research. This could result in recommending such approaches and even having them adopted as effective methods for participatory research or used complementarily for the target group, regardless of cases that impose restrictions. This comparison between the challenges faced when targeting disabled people against the challenges faced when targeting disabled people with the restrictions imposed by the pandemic is not clear, and this is a shortcoming. The comparison could have shown that despite this change being imposed by the pandemic in the end, the new method can help anyway and create better terms for such work even without imposed restrictions. The argument is that it seemed in their modified approach that the remote or virtual activities created a better environment for the group, contradicting their original plan.*

*This would be a distinction that would make the recommendations of this work really valuable to the reader, while now the overall feeling for the lessons learned and recommendations part remain generic and not so impactful.*

 According to your suggestion, we added a new section titled "2.2 Initial challenges of disabled persons participating in transport research“. This section includes the challenges that we predicted in the planning phase of the project with regard to the involvement of people with disabilities in transport research. We also added another section at the end of the paper (3.2 Reflection on the initial challenges and the actual challenges faced in participatory research with persons with disabilities) to compare the initial challenges to the actual challenges when conducting the research, as you recommended. We think this suggestion improved the paper’s focus.

*I would suggest to generally edit the paper in order to use less repetitive and generic language throughout the sections - be more targeted in the descriptions - retouch upon the syntax and grammar, highlight what are the initial challenges and risks in participatory research targeting disabled people and transport, how are these issues being changed by the restrictions imposed for meeting in person, and how the new approaches and the lessons learned while enforcing them enhanced the work or even provided better results and should anyhow be considered for participatory research methods when dealing with disabled people.*

 According to your remarks, we revised the paper and conducted English editing. We also tried to focus our paper more on the specific group of people with disabilities. In line with the suggestions from the other reviewer, we also worked on the discussion section and revised the recommendations in a way that our reflection results are linked to the state of the art and focussed more on the topic of engaging people with disabilities in participatory research in times of the COVID-19 pandemic.

# Peer review: Reviewer response for version 1

**DOI:** 10.21956/openreseurope.15444.r28196

**Published:** 2021-12-22

**Authors:** Vera Köpsel

**Affiliations:** 1 Institute for Marine Ecosystem and Fishery Science (IMF), University of Hamburg, Hamburg, Germany

**Paper Summary**

 The paper under review reflects on alternative stakeholder engagement methodologies used in the face of the COVID-19 pandemic to substitute the originally planned participatory approach of the project. The TRIPS project seeks to co-develop transportation solutions together with mobility-impaired citizens from seven European cities. Due to the social distancing measures in the context of COVID-19, TRIPS had to change its approach to co-creating and evaluating mobility solutions through in-person workshops, brainstorming exercises, and joint design sessions. The paper describes the methods originally envisioned for the project as well as the alternative or adjusted methods applied due to the pandemic. Most of the engagement formats were moved to the digital sphere with online workshops, online peer-to-peer engagement, social media content analysis instead of shadowing sessions, and virtual instead of physical co-design workshops. Each of these alternative methods is described and reflected upon in terms of its suitability and challenges. Subsequently, lessons learned are drawn from the process of adapting the methodology and recommendations are given for fellow researchers in co-creation projects. The paper closes with a short paragraph of conclusions. All in all, the article constitutes a timely case study that will be well-placed in Open Research Europe after undergoing revisions.

**Review Summary**

 In my view, the paper presented here does a fine job of outlining the background problem challenging the TRIPS project and its co-design approach by highlighting the impacts of the COVID-19 pandemic on participatory research projects and citing related and state-of-the-art literature. Recent studies about how the pandemic influences research projects and participatory science are outlined and quoted properly. However, both the introduction and the reflections of the methodology sections have a number of shortcomings that are detailed below. Whilst the description of the challenges for TRIPS and its methodology as well as the adjustments applied to the project outline are interesting to read, I think that a valuable opportunity was missed when the paper was written: a deeper and more meaningful reflection of the particular challenges that TRIPS faced due to the circumstance that it conducted participatory research in with disabled users of transport systems. Focusing more on this facet of the project and the specific challenges it brought to digitalising the co-creation process would have lifted the paper from being nice to being impactful and providing valuable insights to scholars and practitioners working with disabled users in transdisciplinary projects. My recommendation therefore is to spend fewer words on describing the methodologies that were used in general and focusing much more specifically on the adjustments that had to be made when moving the originally planned physical workshops and co-creation exercises to online formats. In my view this shift of focus, along with an overall more critical reflection of the adjustment process and the methods used for online engagement, will give the paper the necessary edge and make it an interesting and valuable contribution to the reflection of transdisciplinary research in times a global pandemic with social distancing restrictions.

**Detailed Review**

 GENERAL:

- The tenses are often mixed throughout the paper. Despite this being a grammatical mistake, it makes the paper harder to read as it is often unclear if the authors are speaking about the originally planned methodology or the new, adjusted methodology. Therefore, please revise the paper with grammar and the tenses in mind and decide whether you would generally like to speak of the TRIPS project in the present or the past tense.
- Also when it comes to the English language *per se*, please revise the paper with regards to spelling and grammar mistakes.
- Please revise the manuscript with particular attention to the inverted commas when it comes to direct quotes.
- I do not know whether this is owed to the format of Open Research Europe, but I found the headings and sub-headings difficult to distinguish. A different formatting or numbering of the different sections would have helped a lot.

INTRODUCTION:

- Pp. 3/4:The introduction is generally well-written and covers current literature on the impact of COVID-19 on participatory research projects. It does, however, not become clear what - against the background of these existing studies - are the research questions of the paper presented here. Clearly stating the goal of the paper in the introduction would help a lot in guiding the reader and clarifying the red thread of this case study.

METHODOLOGY ADJUSTMENTS

- Pp. 7 + Table 1: The methodology of the TRIPS project, both as originally planned as well as adjusted, is outlined in the paper in quite some length. Nonetheless, some points remain unclear to me. What, for example, is precisely meant by peer-to-peer activities? Who are the peers, to whom, and how were they trained for conducting interviews? How were they chosen? As this methodology stands in the centre of the paper, please clarify.
- P. 7 onwards: The paper would generally benefit a lot from a more critical reflection of the adjusted methodologies. Who could / could not be reached due to this changed approach? Did the fact that workshops were held online, for example, make it easier for certain disabled users to participate, but excluded users with other disabilities? It is this detail of reflection that, in my view, would give the paper more depth and an increased the level of information for fellow scholars as well as practitioners working with stakeholders with disabilities.
- P. 7: Considering the social media analysis I also would have expected a deeper reflection. The authors rightfully state that researchers must be trained in conducting social media analyses to successfully complete this part of the project; however, there is no mention of the groups of disabled users that might be excluded from the analysis by focusing on social media content. I am certain that only specific groups of transport users share their experiences on social media, which means that certain other groups are not represented in this analysis at all. Although this might be a fact we might have to live with due to the changed methodology, it certainly deserves some words of reflection.
- P. 10: On page 10, suddenly a method called ‘Sketching’ is mentioned without further explanation. As the focus of this paper lies on methodology, please explain in a sentence or two what this sketching method is and how it was conducted online and/or offline. Was this method originally planned to be used or is it part of the new, adjusted methodology?
- I would have liked to know: Who decided about the adjusted online methodologies? Was that a decision by the central project steering committee or a bottom-up approach?
- P. 9/10: Here it was sometimes unclear to me what was the originally planned and what was the adjustment methodology due to the mix-up of the tenses. Please re-word/edit for clarity.
- As mentioned before, I think a potential strength of this paper could be the reflection of using online methods for engaging with disabled users during (and/or after) the pandemic. This opportunity was not made use of; however, in my view the paper lacks significance if this topic is not moved into focus. Both the recommendations as well as the conclusions currently are not overly surprising or innovative. Many of the recommendations are unspecific and have already been made in other papers. I therefore think that there is a lot to gain here in terms of the usefulness and value of the recommendations - and the paper, generally - if the focus is shifted to the specific challenges posed to a participatory research project during the COVID-19 pandemic when working with disabled users.
- P. 10: A few very general advantages and disadvantages of online engagement are mentioned in the latter part of the paper. These reflections do not go very far, though, and have already been made by other scholars as well. I would recommend to focus more specifically on cooperation with users with disabilities, as this focus would open up the possibility to reflect a lot deeper on specific advantages and disadvantages of online technologies in this context.

LESSONS LEARNED + RECOMMENDATIONS

- P. 10/11 – Recommendations: In the awareness of repeating myself, I once more highlight that the recommendation section of the paper would be a great opportunity to give recommendations specifically to fellow scholars and practitioners who engage with stakeholders with disabilities. With this focus, I believe that the recommendations the paper would be a lot less generic and a lot more surprising and helpful for others in similar situations.
- P. 10: What is meant by ‘mixed presence’? Is that a term for hybrid meetings, online and in person together?
- In the general structure of the paper I am missing a discussion section that goes back to the studies introduced at the start of the paper and setting the TRIPS experiences into the context of existing studies and findings. This does not have to be a lengthy section, but I would find it important to contextualise the experiences the authors made in the TRIPS project against the experiences that others already made. What was similar in your project, what was new? What challenges did you face that were not addressed by other studies yet?
- I really like some of your recommendations which are innovative and add value to the practice of online engagement; for example, I much like the idea of piloting new methodologies in real-life settings before applying them in larger groups. Another really good recommendation is to train co-researchers in certain methods to ensure a good quality of processes and results. Except for those points, though, I suggest to generally revisit the recommendations that you give to make them more meaningful and specific to the context that you work in. Practices such as adapting workshops to local contexts and local languages are not a novel approach arising in the face of COVID-19, but should be common practice among researchers who engage with non-academic actors. The recommendation of staying connected with your stakeholders and being flexible in the methodological approach are generic and have been made in several studies already (including mine). I am aware that it is difficult at this stage to give genuinely helpful new recommendations, but I really think that focusing your recommendations on the specific circumstances of your project would add a lot of value here.

All in all I would like to thank the authors for submitting this paper and sharing their experiences. I recommend to Open Research Europe to approve the paper with reservations. A shift of the currently rather generic topic of the case study to the more specific circumstances under which the TRIPS project worked would sharpen the focus of the article and add a lot of value to its conclusions and recommendations. After undergoing revisions and language corrections, though, I am curious to read the final version of the paper and learn more about online engagement with stakeholders with disabilities.

Is the case presented with sufficient detail to be useful for teaching or other practitioners?

Partly

Is the work clearly and accurately presented and does it cite the current literature?

Yes

If applicable, is the statistical analysis and its interpretation appropriate?

Yes

Are all the source data underlying the results available to ensure full reproducibility?

Yes

Are the conclusions drawn adequately supported by the results?

Yes

Is the background of the case’s history and progression described in sufficient detail?

Yes

Reviewer Expertise:

Stakeholder engagement in EU projects and transdisciplinary research; coordination of stakeholder engagement; online and offline engagement methods; challenges of stakeholder engagement during COVID-19; human geography; social scientific methods

I confirm that I have read this submission and believe that I have an appropriate level of expertise to confirm that it is of an acceptable scientific standard, however I have significant reservations, as outlined above.

# Response

**Published:** 2022-03-14

**Authors:** Alexandra König

**Affiliations:** German Aerospace Center (DLR), Germany

- *I think that a valuable opportunity was missed when the paper was written: a deeper and more meaningful reflection of the particular challenges that TRIPS faced due to the circumstance that it conducted participatory research in with disabled users of transport systems. Focusing more on this facet of the project and the specific challenges it brought to digitalising the co-creation process would have lifted the paper from being nice to being impactful and providing valuable insights to scholars and practitioners working with disabled users in transdisciplinary projects. My recommendation therefore is to spend fewer words on describing the methodologies that were used in general and focusing much more specifically on the adjustments that had to be made when moving the originally planned physical workshops and co-creation exercises to online formats. In my view this shift of focus, along with an overall more critical reflection of the adjustment process and the methods used for online engagement, will give the paper the necessary edge and make it an interesting and valuable contribution to the reflection of transdisciplinary research in times a global pandemic with social distancing restrictions.*

We agree with your suggestion that the paper will improve by providing more information on the challenges that the project faced due to the fact that it focuses on people with disabilities. We thus added a new section to the introduction that stands under the title: "Initial challenges of disabled persons participating in transport research“. We listed here five key challenges that we planned to face during the participatory process of engaging people with disabilities. We also added a new section to the discussion called "Reflection on the initial challenges and the actual challenges faced in participatory research with persons with disabilities“ to discuss and contrast the initial and the actual challenges faced.  

- *The tenses are often mixed throughout the paper. Despite this being a grammatical mistake, it makes the paper harder to read as it is often unclear if the authors are speaking about the originally planned methodology or the new, adjusted methodology. Therefore, please revise the paper with grammar and the tenses in mind and decide whether you would generally like to speak of the TRIPS project in the present or the past tense.*
- *Also when it comes to the English language per se, please revise the paper with regards to spelling and grammar mistakes.*
- *Please revise the manuscript with particular attention to the inverted commas when it comes to direct quotes.*

As suggested, we revised the manuscript with regard to English language. This was done by a native speaker.

- *I do not know whether this is owed to the format of Open Research Europe, but I found the headings and sub-headings difficult to distinguish. A different formatting or numbering of the different sections would have helped a lot.*

We agree that the clarity of the paper would improve by using a numbering of the sections. We did so and added numbers.

*INTRODUCTION:*

- *Pp. 3/4:The introduction is generally well-written and covers current literature on the impact of COVID-19 on participatory research projects. It does, however, not become clear what - against the background of these existing studies - are the research questions of the paper presented here. Clearly stating the goal of the paper in the introduction would help a lot in guiding the reader and clarifying the red thread of this case study.*

We absolutely agree and added some paragraphs regarding the research gap and the addressed research question to the end of the introduction.

*METHODOLOGY ADJUSTMENTS*

- *Pp. 7 + Table 1: The methodology of the TRIPS project, both as originally planned as well as adjusted, is outlined in the paper in quite some length. Nonetheless, some points remain unclear to me. What, for example, is precisely meant by peer-to-peer activities? Who are the peers, to whom, and how were they trained for conducting interviews? How were they chosen? As this methodology stands in the centre of the paper, please clarify.*

Thank you for this remark. We added some more information concerning the team building and the training of the working group members. Please see section 2.3. We also clarified the term 'peer‘.

- *P. 7 onwards: The paper would generally benefit a lot from a more critical reflection of the adjusted methodologies. Who could / could not be reached due to this changed approach? Did the fact that workshops were held online, for example, make it easier for certain disabled users to participate, but excluded users with other disabilities? It is this detail of reflection that, in my view, would give the paper more depth and an increased the level of information for fellow scholars as well as practitioners working with stakeholders with disabilities.*

We appreciate your comment and think it is valuable. However, the critical reflection of the adjusted methodologies is not part of the method description in section 2 but part of section 3 ('Discussion‘). We added some more thoughts regarding this topic in the section '3.2 Reflection on the initial challenges and the actual challenges faced in participatory research with persons with disabilities **.'** We hope, you find the reflection of the adjusted methodologies in a good shape now.

  

- *P. 7: Considering the social media analysis I also would have expected a deeper reflection. The authors rightfully state that researchers must be trained in conducting social media analyses to successfully complete this part of the project; however, there is no mention of the groups of disabled users that might be excluded from the analysis by focusing on social media content. I am certain that only specific groups of transport users share their experiences on social media, which means that certain other groups are not represented in this analysis at all. Although this might be a fact we might have to live with due to the changed methodology, it certainly deserves some words of reflection.*

We are thankful for this comment. We followed your suggestion and added some more information regarding the limitations according to the exclusion of specific groups of disabled users to the section. We also added a literature reference for this.

- *P. 10: On page 10, suddenly a method called ‘Sketching’ is mentioned without further explanation. As the focus of this paper lies on methodology, please explain in a sentence or two what this sketching method is and how it was conducted online and/or offline. Was this method originally planned to be used or is it part of the new, adjusted methodology?*

Thank you for this remark. We restructured the text and added more information and an exemplary sketch to visualize the method. Please see section 2.3.

- *I would have liked to know: Who decided about the adjusted online methodologies? Was that a decision by the central project steering committee or a bottom-up approach?*

We think this is a very interesting remark. We decided to add some information on the decision process to the paper. Please see section 2.3.

- *P. 9/10: Here it was sometimes unclear to me what was the originally planned and what was the adjustment methodology due to the mix-up of the tenses. Please re-word/edit for clarity.*

We checked the tenses according to your suggestion.

- *As mentioned before, I think a potential strength of this paper could be the reflection of using online methods for engaging with disabled users during (and/or after) the pandemic. This opportunity was not made use of; however, in my view the paper lacks significance if this topic is not moved into focus. Both the recommendations as well as the conclusions currently are not overly surprising or innovative. Many of the recommendations are unspecific and have already been made in other papers. I therefore think that there is a lot to gain here in terms of the usefulness and value of the recommendations - and the paper, generally - if the focus is shifted to the specific challenges posed to a participatory research project during the COVID-19 pandemic when working with disabled users.*

We agree and adapted the methodology reflection according to your suggestion to highlight the changes due to using online methods for people with disabilities. We added more information regarding this topic in section 3.1. We also followed your recommendation and tried to shorten the text where possible.

- *P. 10: A few very general advantages and disadvantages of online engagement are mentioned in the latter part of the paper. These reflections do not go very far, though, and have already been made by other scholars as well. I would recommend to focus more specifically on cooperation with users with disabilities, as this focus would open up the possibility to reflect a lot deeper on specific advantages and disadvantages of online technologies in this context.*

The previous point also addresses this suggestion. Please see the revised section 3.1.

*LESSONS LEARNED + RECOMMENDATIONS*

- *P. 10/11 – Recommendations: In the awareness of repeating myself, I once more highlight that the recommendation section of the paper would be a great opportunity to give recommendations specifically to fellow scholars and practitioners who engage with stakeholders with disabilities. With this focus, I believe that the recommendations the paper would be a lot less generic and a lot more surprising and helpful for others in similar situations.*

We appreciate your comment regarding the specificity of the recommendations. We followed your suggestion and described the recommendations in more detail and with a stronger fit to disabled users.  

- *P. 10: What is meant by ‘mixed presence’? Is that a term for hybrid meetings, online and in person together?*

We clarified this and provided more information on the topic.

- *In the general structure of the paper I am missing a discussion section that goes back to the studies introduced at the start of the paper and setting the TRIPS experiences into the context of existing studies and findings. This does not have to be a lengthy section, but I would find it important to contextualise the experiences the authors made in the TRIPS project against the experiences that others already made. What was similar in your project, what was new? What challenges did you face that were not addressed by other studies yet?*

We are thankful for this comment. We revised the discussion section and refered to the references presented in the state of the art. We further worked out additional recommendations that came out of our reflection more clearly.

- *I really like some of your recommendations which are innovative and add value to the practice of online engagement; for example, I much like the idea of piloting new methodologies in real-life settings before applying them in larger groups. Another really good recommendation is to train co-researchers in certain methods to ensure a good quality of processes and results. Except for those points, though, I suggest to generally revisit the recommendations that you give to make them more meaningful and specific to the context that you work in. Practices such as adapting workshops to local contexts and local languages are not a novel approach arising in the face of COVID-19, but should be common practice among researchers who engage with non-academic actors. The recommendation of staying connected with your stakeholders and being flexible in the methodological approach are generic and have been made in several studies already (including mine). I am aware that it is difficult at this stage to give genuinely helpful new recommendations, but I really think that focusing your recommendations on the specific circumstances of your project would add a lot of value here.*

We see your point and revised the recommendations accordingly to your suggestion. We deleted one of the recommendations and enriched the others by giving more detailed information and references.
